# Dual Role of CREB in The Regulation of VSMC Proliferation: *Mode of Activation Determines Pro- or Anti-Mitogenic Function*

**DOI:** 10.1038/s41598-018-23199-4

**Published:** 2018-03-20

**Authors:** Claire Hudson, Tomomi E. Kimura, Aparna Duggirala, Graciela B. Sala-Newby, Andrew C. Newby, Mark Bond

**Affiliations:** 1 Translational Health Sciences, University of Bristol, Research Floor Level 7, Bristol Royal Infirmary, Bristol, BS2 8HW UK; 2 Present Address: School of Life Sciences, University of Warwick, Coventry, CV4 7AL UK

## Abstract

Vascular smooth muscle cell (VSMC) proliferation has been implicated in the development of restenosis after angioplasty, vein graft intimal thickening and atherogenesis. We investigated the mechanisms underlying positive and negative regulation of VSMC proliferation by the transcription factor cyclic AMP response element binding protein (CREB). Incubation with the cAMP elevating stimuli, adenosine, prostacyclin mimetics or low levels of forksolin activated CREB without changing CREB phosphorylation on serine-133 but induced nuclear translocation of the CREB co-factors CRTC-2 and CRTC-3. Overexpression of CRTC-2 or -3 significantly increased CREB activity and inhibited VSMC proliferation, whereas CRTC-2/3 silencing inhibited CREB activity and reversed the anti-mitogenic effects of adenosine A2B receptor agonists. By contrast, stimulation with serum or PDGF_BB_ significantly increased CREB activity, dependent on increased CREB phosphorylation at serine-133 but not on CRTC-2/3 activation. CREB silencing significantly inhibited basal and PDGF induced proliferation. These data demonstrate that cAMP activation of CREB, which is CRTC2/3 dependent and serine-133 independent, is anti-mitogenic. Growth factor activation of CREB, which is serine-133-dependent and CRTC2/3 independent, is pro-mitogenic. Hence, CREB plays a dual role in the regulation of VSMC proliferation with the mode of activation determining its pro- or anti-mitogenic function.

## Introduction

Vascular smooth muscle cell (VSMC) proliferation and migration contributes towards the development of various vascular diseases characterised by pathological intima formation, including atherosclerosis, transplant vasculopathy and pulmonary hypertension. Increased VSMC proliferation and migration also contributes towards restenosis after angioplasty and late vein graft failure. Interruption of this cycle can slow disease progression and limit pathological intima formation. This is exemplified by medications that lower low density lipoprotein (LDL) cholesterol or block cell proliferation, which have revolutionized preventive therapy for cardiovascular disease (CVD) and ameliorated the problem of angioplasty restenosis, respectively. However, these do not fully normalize CV risk and additional treatments are therefore needed.

The second messenger 3′-5′ cyclic adenosine monophosphate (cAMP) is synthesized in cells by adenylyl cyclase enzymes in response to vasoactive G_s_ protein-coupled receptor stimulation. In VSMC, elevated cAMP inhibits mitogen stimulated proliferation *in vitro* and after injury *in vivo*^1–5^. Inhibitory effects of cAMP on VSMC migration also help reduce intimal lesion formation in experimental vascular injury models^6,7^. Moreover, altered or aberrant cAMP signalling is implicated in the development of many vascular pathologies^8,9^, including late vein-graft failure^10–12^, angioplasty restenosis^13^ cardiac hypertrophy^14^, atherogenesis^15^ and pulmonary hypertension. For example, the ability of VSMC to synthesise cAMP is reduced in response to hypertension^16^, vein grafting^12^, hypoxia and oxLDL^17^. cAMP levels are also reduced in blood mononuclear cells in CVD patients^18^. This suggests that deregulation of cAMP signalling is a common feature of many vascular diseases. Moreover, stimulation of cAMP signalling has numerous beneficial effects including reductions in neointima formation^7,19^, VSMC migration^20^, VSMC proliferation^1,19^, endothelial permeability^21^, inflammation, ROS generation, plasma triglycerides and monocyte recruitment. Therefore, treatments that elevate or mimic the action of cAMP carry significant therapeutic promise because they improve endothelial function, inhibit VSMC cell proliferation and migration and reduce inflammation.

The anti-mitogenic effects of cAMP are mediated via activation of cAMP sensitive Protein Kinase A (PKA) and Exchange Protein Activated by cAMP (EPAC), which synergise to regulate levels of several cell-cycle proteins^2,22^. However, the mechanisms downstream of PKA and EPAC are incompletely characterised. Activation of the cAMP sensitive transcription factor CREB may represent one important mechanism underlying cAMP-dependent cellular responses. Some studies have suggested anti-mitogenic properties of CREB in VSMC. For example, CREB levels are high in differentiated VSMC but decrease during phenotypic modulation^20^. Stimuli that enhance cAMP-signalling and inhibit VSMC proliferation and are associated with increased CREB activity^22,23^. Consistent with this, CREB inhibition with siRNA or dominant-negative mutants is sufficient to induce VSMC proliferation^20,24,25^, whereas overexpression of constitutively-active mutants of CREB arrests cell-cycle progression^20,23,25^. However, several other studies have reported pro-mitogenic properties of CREB in VSMC. For example, VSMC proliferation induced by angiotensin II^26^, thrombin^27^ or the interferon-γ inducible protein-10^28^ is dependent on CREB activation. These observations suggest that CREB activation may play a role in both mitogenic and anti-mitogenic responses in VSMC.

Phosphorylation of CREB at serine-133 is one important mechanism of CREB-mediated transcriptional activation^29^. PKA is implicated since this residue is part of a consensus PKA phosphorylation site (RXXS) and its phosphorylation can be blocked by PKA inhibition^30^. However, selective PKA activation fails to inhibit VSMC proliferation^2^, suggesting that PKA-mediated phosphorylation of CREB at serine-133 may not be sufficient for the anti-mitogenic effects of cAMP in these cells. Furthermore, several studies have also suggested that phosphorylation of CREB at serine-133 may not be essential for CREB-dependent gene expression, implicating alternative mechanisms. For example, T-cell receptor stimulation of Jurkat cells induces high levels of CREB serine-133 phosphorylation without inducing target gene expression^31^. Induction of CREB activity by voltage-sensitive Ca^2+^ channels is markedly reduced in a PKA-deficient PC12 cells, despite robust CREB phosphorylation at serine-133^32^. Expression of mouse hippocampal CREB target genes in response to fear conditioning is unaffected in CREB-S133A transgenic mice^33^. Importantly, CREB can be activated independently of serine-133 phosphorylation by binding of CREB Regulated Transcription Coactivators (CRTC1, CRTC2 or CRTC3)^34^. However, the role of CRTC-regulated CREB activity in VSMC is unknown. Here, we investigated the relative importance of CREB serine-133 phosphorylation and nuclear translocation of CRTCs in pro- and anti-mitogenic effects on VSMCs.

## Results

### Forskolin and GPCR agonists induce CREB-dependent transcription independently of changes in CREB serine-133 phosphorylation

**Figure 1:**
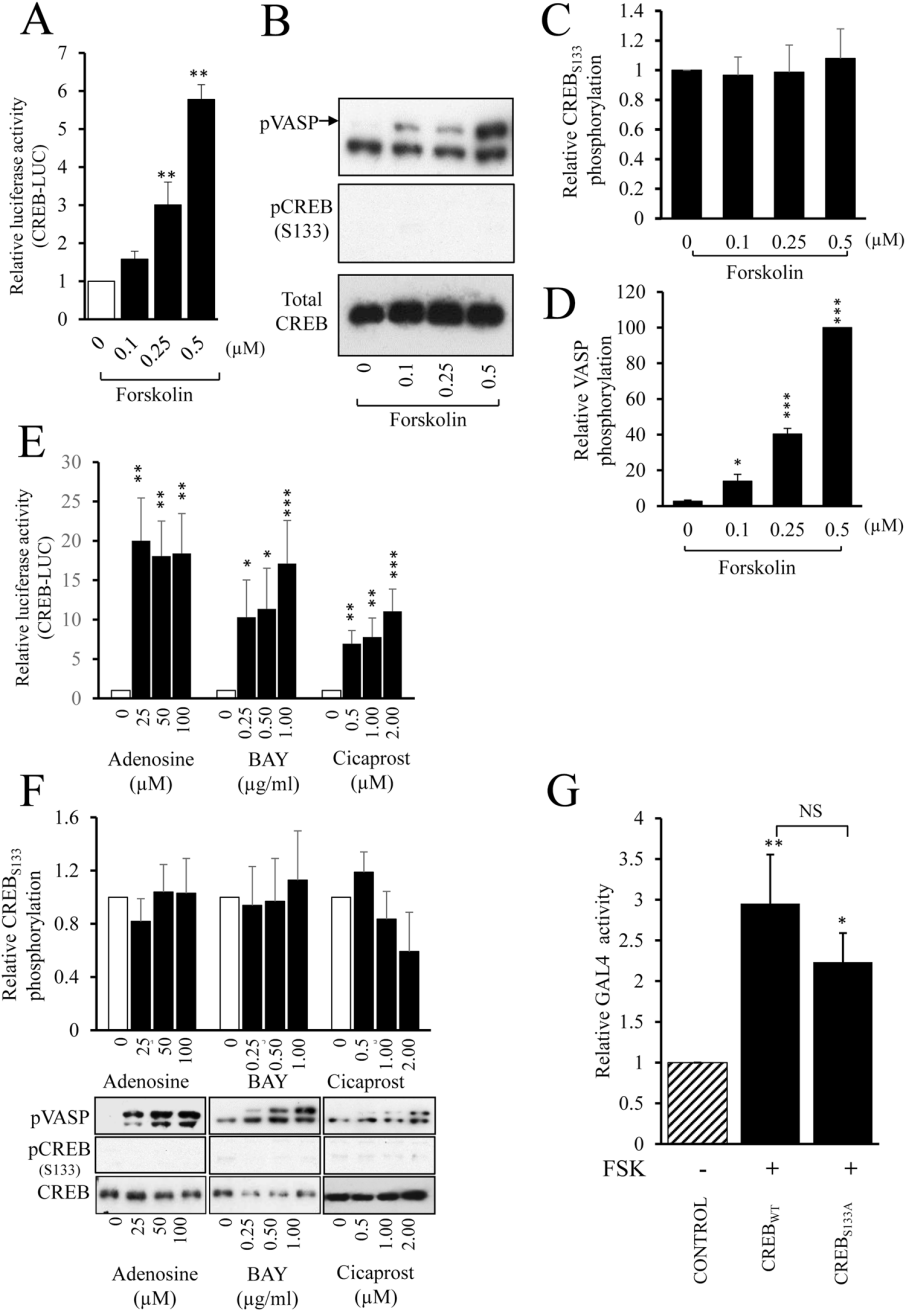
Forskolin and GPCR agonists induce CREB-dependent transcription independently of changes in CREB serine-133 phosphorylation. VSMC (n = 3) were transfected with CREB-LUC, serum starved for 4 h before being stimulated for 4 h with indicated doses of forskolin and assayed for luciferase activity (**A**). VSMC (n = 5) were serum starved for 4 h before being treated with forskolin for 30 min; cell lysates were analysed for phospho-VASP, phospho-CREB and total CREB by western blotting (**B**) and densitometric analysis (**C**; n = 5 and **D**; n = 4). VSMC were transfected with CREB-LUC, serum starved for 4 h before being stimulated for 4 h with indicated concentrations of adenosine (n = 4), BAY60-6583 (BAY, n = 6) or Cicaprost (n = 6) and assayed for luciferase activity (**E**). VSMC lysates were analysed by western blotting for phospho-VASP, pCREB and total CREB (**F**). VSMC were transfected with GAL4-NLUC together with either a GAL4, GAL4-CREB_wild-type_ or GAL4-CREB_S133A_ expression vector. Cells were serum starved for 4 hours before stimulation with 0.5 µM forskolin for 4 hours and subsequent nano-luciferase measurement (**G**, n = 5). *Indicates p < 0.05, **indicates p < 0.01, ***indicates p < 0.001 relative to control; one-way repeated measures ANOVA with Student Newman Keuls post-hoc tests.

We initially investigated the effect of elevated cAMP levels on CREB-dependent gene expression and serine-133 phosphorylation using the adenylate cyclase activator forskolin. Stimulation of VSMC with low doses of forskolin (0.25–0.5 µM) significantly induced CREB-dependent reporter gene activity after 4 hours (Fig. 1A), whereas 0.1 µM forskolin did not significantly increase CREB activity. However, activation of CREB reporter activity by forskolin was not associated with an increase in CREB phosphorylation at serine-133 (Fig. 1B and C), despite robust PKA activation detected by increased phosphorylation of VASP (Fig. 1B and D). Since other studies have reported that forskolin induces serine-133 phosphorylation of CREB, we investigated stimulation with a supraphysiological dose of forskolin (25 µM) and confirmed that this did increase CREB phosphorylation at serine-133 (Supplement Fig. 1). We also verified that 0.5 µM FSK did not increase CREB phosphorylation over a time course from 5 to 120 minutes (Supplementary Figure 1). We next tested whether elevation of cAMP levels in response to physiological stimulation of adenosine A2B and prostacyclin receptors also induces CREB-dependent transcription independently of changes in CREB serine-133 phosphorylation. Stimulation of VSMC with adenosine, the adenosine-A2B receptor agonist BAY60-6583 or the prostacyclin receptor agonist Cicaprost significantly increased CREB-dependent reporter gene activity after 4 hours (Fig. 1E). However, activation of CREB-dependent transcription by any of these agonists did not increase CREB phosphorylation at serine-133 (Fig. 1F), despite robust phosphorylation of VASP. To verify that adenosine, BAY60-6583 or Cicaprost were unable to increase CREB phosphorylation at earlier time points, a detailed time course was conducted (Supplementary Figure 3). To confirm that elevated cAMP is able to activate CREB independently of serine-133 phosphorylation we tested the ability of forskolin to stimulate the activity of a GAL4 reporter gene in cells expressing either GAL4-CREB_wild-type_ or GAL4-CREB_S133A_, in which serine-133 is mutated to an alanine. Stimulation with 0.5 µM forskolin significantly induced GAL4 reporter activity in cells expressing GAL4-CREB_wild-type_ or GAL4-CREB_S133A_ to a similar extent (Fig. 1G). Taken together, these data imply that elevation of cAMP, either via activation of adenosine A2B or prostacyclin receptors or via adenylate cyclase activation with low concentrations of forskolin, induces CREB-dependent transcription independently of changes in CREB serine-133 phosphorylation.

### PKA and EPAC act synergistically to activate CREB independently of changes in CREB serine-133 phosphorylation

**Figure 2:**
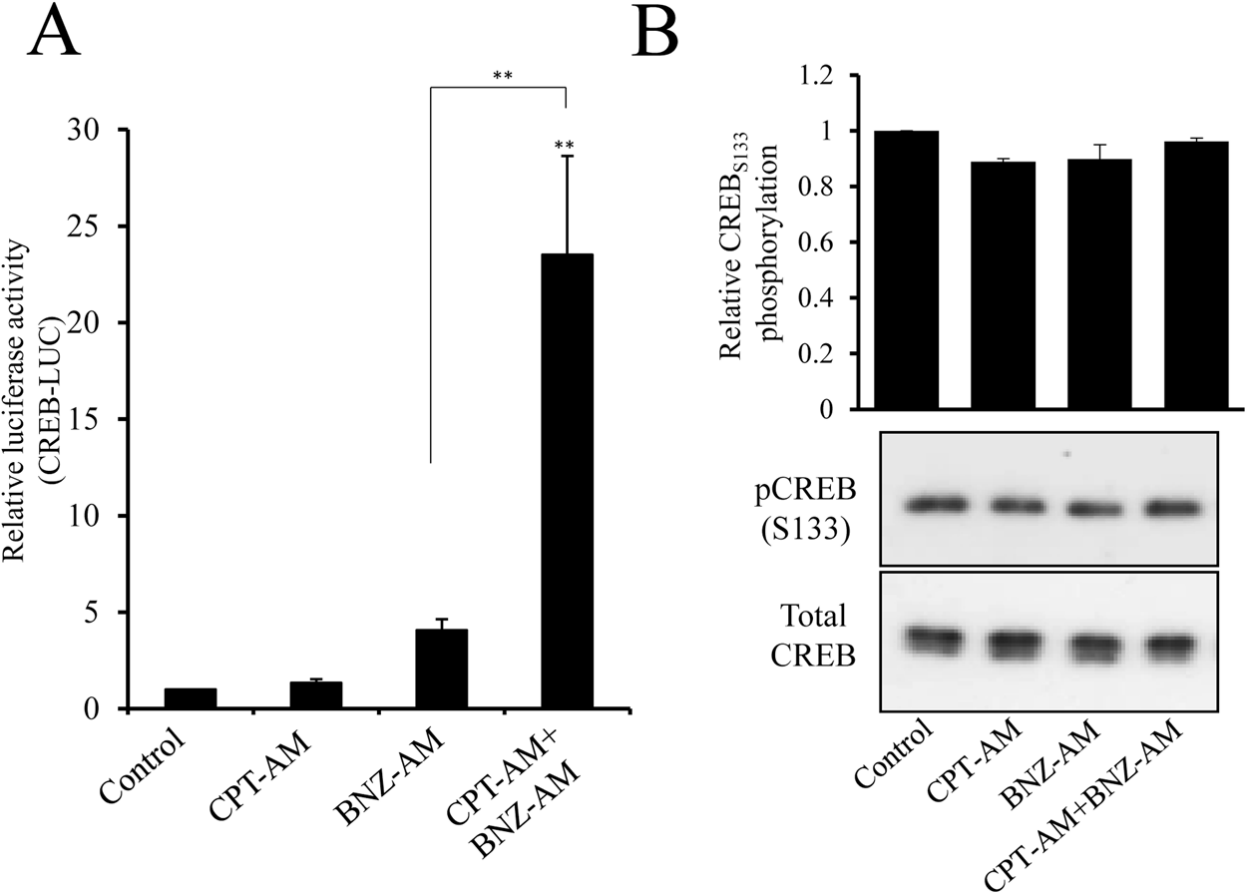
PKA and EPAC act synergistically to activate CREB independently of changes in CREB serine-133 phosphorylation. VSMC were transfected with CREB-LUC, serum starved for 4 h before being stimulated for 2 h with 2 µM 8-CPT-2′-O-Me-cAMP-AM (CPT) to activate EPAC, 4 µM 6-Bnz-cAMP-AM (BNZ) to activate PKA, or both, then assayed for luciferase activity (n = 3; **A**). VSMC were serum starved for 4 hours before being treated with the CPT and/or BNZ for 30 min. Cell lysates were used for western blotting and densitometric analysis with pCREB and total CREB antibodies (n = 3; **B**).

We previously demonstrated that the anti-mitogenic properties of cAMP in VSMC are dependent on signalling via both PKA and EPAC^2^. 6-BNZ-cAMP-AM (BNZ-AM) is an extremely membrane-permeant precursor of the specific PKA agonist 6-BNZ-cAMP. Selective activation of PKA by this agonist has been validated previously^2,35^. 8-pCPT-2′-O-Me-cAMP-AM (CPT-AM) is a membrane-permeant precursor of the specific EPAC agonist 8-pCPT-2′-O-Me-cAMP. Selective activation of EPAC but not PKA by this agonist has been validated previously^2,36^. In this study, stimulation with BNZ-AM or CPT-AM alone did not significantly increase CREB activity (Fig. 2A). However, CREB activity was significantly increased after 2 hours stimulation with BNZ-AM plus CPT-AM (Fig. 2A, p < 0.01), suggesting that PKA and EPAC act synergistically to induce CREB-dependent transcription. Importantly, selective activation of PKA, EPAC or PKA plus EPAC did not induce any increase in phosphorylation of CREB at serine-133 (Fig. 2B).

### Forskolin and GPCR agonists induce nuclear translocation of CRTC2 and CRTC3 via PKA and EPAC

**Figure 3:**
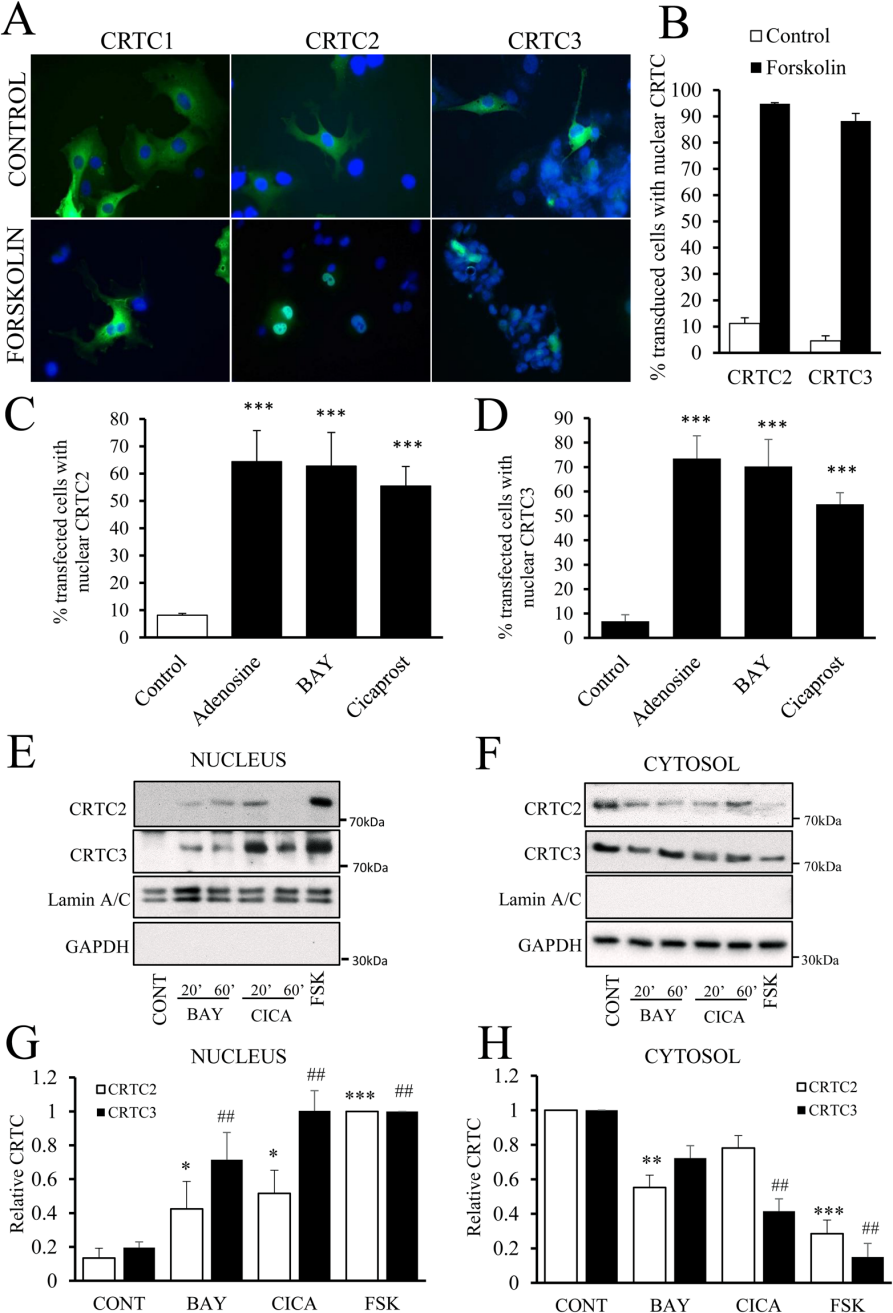
Forskolin and GPCR agonists induce nuclear translocation of CRTC2 and CRTC3. VSMC were transfected with GFP-CTRC1, 2 or 3 by electroporation and after 24 h were serum starved for 24 h. Cells were stimulated for 1 h with 0.5 µM forskolin (**A** and **B**, n = 3), 100 µM adenosine (n = 4; **C**,**D**), 5 µg/ml BAY60-6583 (BAY, n = 4; **C**,**D**), or 1 µM Cicaprost (n = 4; **C**,**D**). Cells were analysed for cellular localisation of CRTC proteins by fluorescence microscopy and image analysis. Data were expressed as % of cells with predominantly nuclear CRTC protein. VSMC (n = 6) serum starved for 4 h were treated for 20 or 60 min with 5 µg/ml BAY, 1 µM Cicaprost (CICA) or 10 µM forskolin (FSK). Cells were subjected to cytosolic and nuclear fractionation and proteins were subjected to SDS-PAGE and western blotting for CRTC2, CRTC3, Lamin A/C and GAPDH (**E** and **F**). CRTC2 and CRTC3 levels were quantified by densitometry and data were expressed relative to FSK (nucleus, **G**) or CONT (cytosol, **H**). *Indicates p < 0.05, ***indicates p < 0.001 relative to control; one-way repeated measures ANOVA with Student Newman Keuls post-hoc tests.

**Figure 4:**
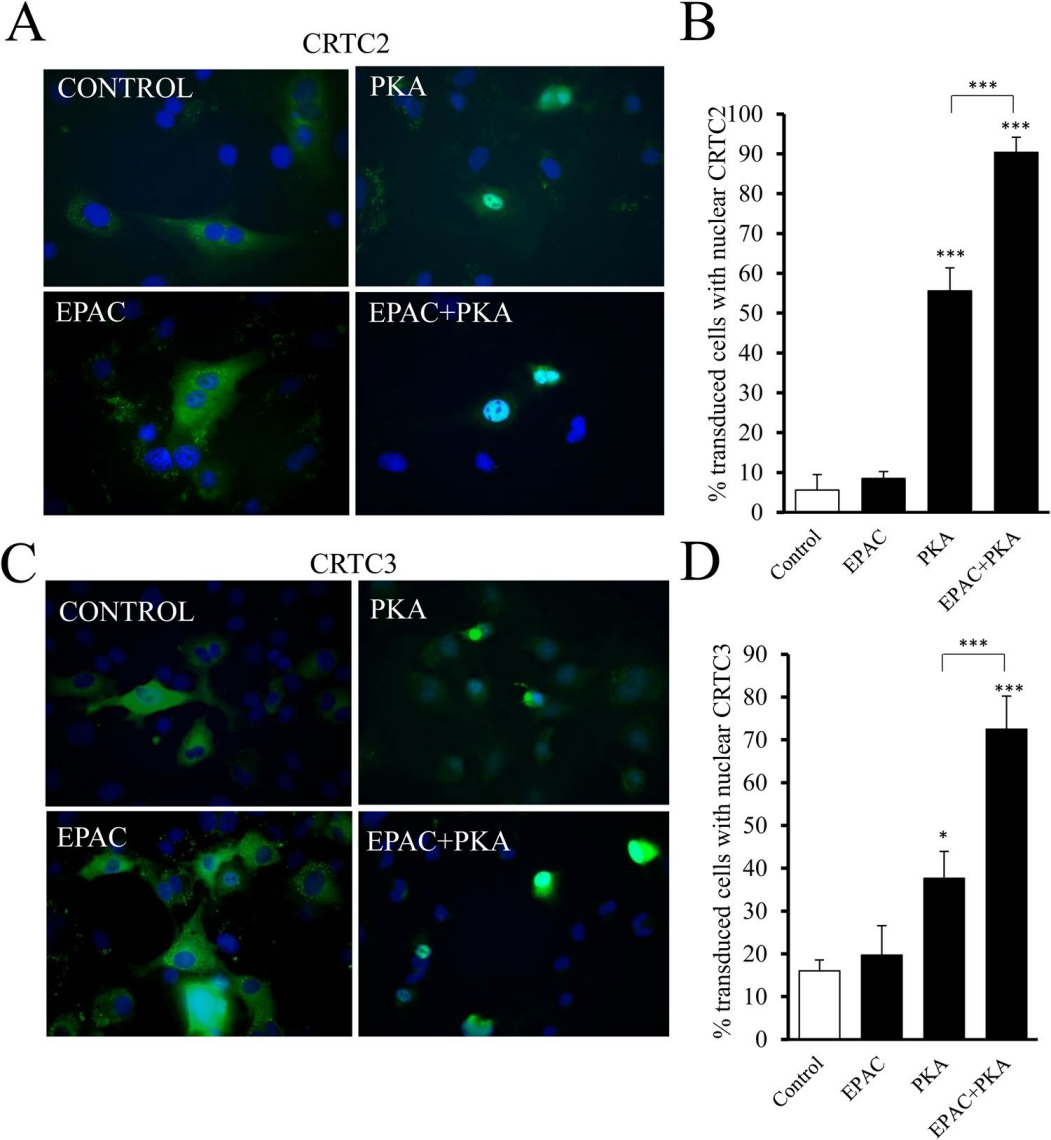
PKA and EPAC act synergistically to induce nuclear translocation of CRTC2 and CRTC3. VSMC were transfected with GFP-CRTC 2 (n = 4; **A** and **B**) or GFP-CRTC3 (**C** and **D**) by electroporation and serum starved for 24 h. Cells were stimulated for 1 h with 2 µM 8-CPT-2′-O-Me-cAMP-AM to activate EPAC, 4 µM 6-Bnz-cAMP-AM to activate PKA or both together. Cells were analysed for cellular localisation of CRTC proteins by fluorescence microscopy and image analysis. Data were expressed as % of transfected cells with nuclear CRTC2 (**B**) or CRTC3 (n = 3; **D**) protein. *Indicates p < 0.05, ***indicates p < 0.001 relative to control; one-way repeated measures ANOVA with Student Newman Keuls post-hoc tests.

We investigated whether forskolin or GPCR agonist-mediated activation of CREB-dependent transcription is mediated by the CREB Regulated Transcription Coactivators (CRTC1, CRTC2 and CRTC3). In plasmid transduced, serum starved cells, GFP-tagged CRTC1, CRTC2 and CRTC3 were all predominantly cytoplasmic (Fig. 3A). CRTC1 remained cytoplasmic after forskolin stimulation. However, CRTC2 and CRTC3 both re-distributed to the nucleus within 1 hour of forskolin stimulation (Fig. 3A and B). We therefore focused our attention on CRTC2 and CRTC3. Activation of adenosine A2B receptors with adenosine or BAY60-6583 and prostacyclin receptors with Cicaprost also resulted in a significant nuclear translocation of CRTC2 and 3 (Fig. 3C and D). We next used cellular fractionation to confirm that these stimuli induce a similar nuclear translocation of endogenous CRTC2 and CRTC3 proteins. Separation of cytosolic and nuclear proteins was effective, as demonstrated by the exclusive localisation of GAPDH and Lamin A/C in these respective fractions (Fig. 3E and F). Stimulation with BAY60-6583 or Cicaprost resulted in a rapid (within 20 minutes) nuclear translocation of CRTC2 and CRTC3 (Fig. 3E–H). High dose forskolin (10 µM) was included as a positive control and to aid quantification. We next stimulated cells with the PKA and EPAC selective agonists, BNZ-AM or CPT-AM, to test if these cAMP effectors regulate CREB activity by cooperating to induce CRTC nuclear translocation. Selective activation of EPAC, with CPT-AM did not significantly induce nuclear localisation of CRTC2 or CRTC3. In contrast, selective activation of PKA with BNZ-AM resulted in a significant but sub-maximal induction of nuclear localisation of CRTC2 and CRTC3. Importantly, this was significantly enhanced by co-activation of EPAC, indicating a synergistic action of these proteins on CRTC2 and CRTC3 activation (Fig. 4A–D).

### CRTC2 and CRTC3 activate CREB-independently of serine-133 phosphorylation and inhibit VSMC proliferation

**Figure 5:**
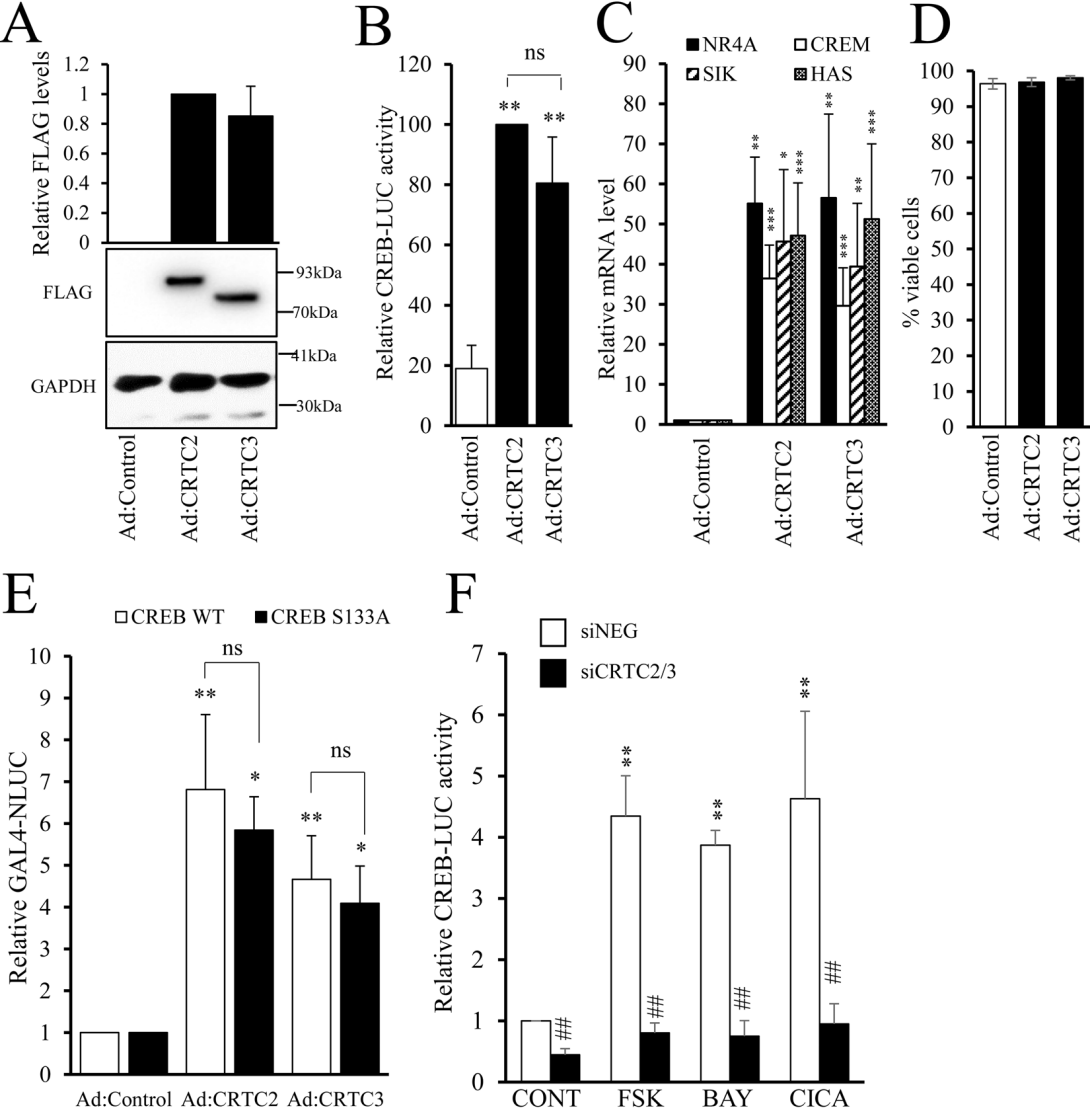
CRTC2 or CRTC3 mediate cAMP induced CREB activity. VSMC were infected with control adenovirus (Ad:control) or adenovirus expressing active-CRTC2 (Ad:CRTC2) or active-CRTC3 (Ad:CRTC3) and equal expression verified by western blotting with an anti-FLAG antibody (**A**, n = 4). Cells were transfected with CREB-LUC and 24 h later infected with CRTC or control adenovirus, luciferase activity was measured in cell lysates after 24 h (**B**, n = 4). mRNA levels of NR4A, CREM, SIK and HAS were assessed in Ad:CRTC2 or 3 infected cells by RT-qPCR (**C**, n = 4), and cell viability determined using trypan blue after 48 h viral expression (**D**, n = 4). VSMC were transfected with GAL4-NLUC together with either a GAL4, GAL4-CREB_wild-type_ or GAL4-CREB_S133A_ expression vector. Cells were infected with adenoviruses expressing constitutively active CRTC2 or CRTC3 and GAL4-NLUC activity quantified 24 hours later (**E**). VSMC were co-transfected with CREB-LUC reporter and scrambled siRNA (siNEG) or siRNA against CRTC2 and CRTC3 (siCRTC2/3). After 48 hours, cells were serum starved for 4 h before being treated with forskolin (FSK, 0.5 µM; n = 3), BAY60-6583 (BAY, 1 µg/ml; n = 3) or Cicaprost (CICA, 1 µM; n = 3) for 4 h. Cells were lysed for luciferase measurement (**F**). Data were analysed with one-way ANOVA with Student Newman Keuls post-tests; **indicates p < 0.01 relative to control, ^##^indicates p < 0.01 relative to siNEG.

Elevated cAMP has well documented anti-mitogenic effects in VSMC^1,2,19,22^. We therefore investigated the role of cAMP/CRTC-dependent CREB activation on VSMC proliferation and migration. We utilised three approaches to activate and inhibit CRTCs: adenovirus mediated expression of constitutively-active CRTC2 (Ad:CRTC2_S171A_) or CRTC3 (Ad:CRTC3_S161A_); pharmacological inhibition of Salt Inducible Kinase (SIK), which phosphorylates CRTC and represses activation; and siRNA-mediated silencing of CRTC2 and CRTC3. Infection of VSMC with Ad:CRTC2_S171A_ or Ad:CRTC3_S161A_ resulted in equal expression of FLAG-tagged CRTC2 and CRTC3 protein (Fig. 5A) and a significant increase in CREB activity (Fig. 5B). Expression of active CRTC2 and CRTC3 also stimulated the expression of the classical CREB target genes *Nr4A*, *Crem*, *Sik* and *Has* (Fig. 5C) without altering cell viability (Fig. 5D). Active CRTC2 and CRTC3 also stimulated the activity of a GAL4-reporter in cells expressing GAL4-CREB_wild-type_ and this was not significantly different in cells expressing GAL4-CREB_S133A_ (Fig. 5E). Furthermore, dual silencing of both CRTC2 and CRTC3 prevented induction of CREB activity in response to forskolin, BAY60-6583 or Cicaprost (Fig. 5F). These data indicate that activation of CRTC2 and CRTC3 is responsible for CREB activation in response to cAMP-elevating stimuli in VSMC and that this occurs independently of CREB serine-133 phosphorylation.

**Figure 6:**
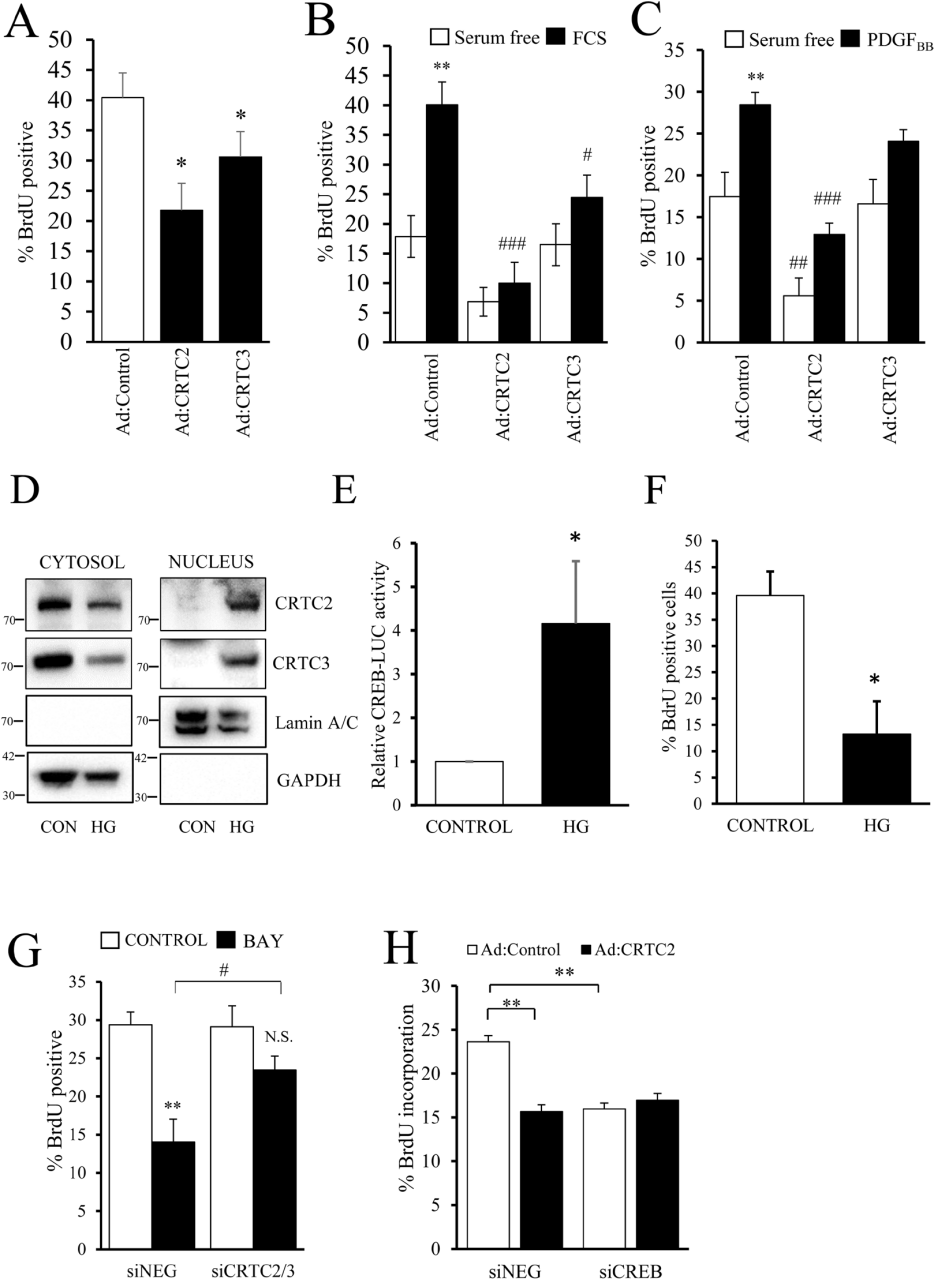
Expression of active CRTC2/3 inhibits VSMC proliferation but not migration, and CRTC2/3 contribute to cAMP-induced inhibition of proliferation. Asynchronously proliferating cells in 5% FCS were infected with control adenovirus (Ad:control) or adenovirus expressing active-CRTC2 (Ad:CRTC2) or active-CRTC3 (Ad:CRTC3) for 24 h and incubated for a further 6 h with 10 µM BrdU to label proliferating cells (**A**, n = 4). VSMC expressing active CRTC2 or CRTC3 were serum starved for 24 h and proliferation was stimulated with 5% FCS (**B**, n = 5) or 25 ng/ml PDGF_BB_ (**C**, n = 7) for 24 h, with BdrU present during the final 6 h. Serum-starved VSMC were stimulated with 2 µM HG-9-91-01 for 45 min and nuclear translocation of endogenous CRTC2 and CRTC3 was analysed by cell fractionation and western blotting (**D**). VSMC were transfected with CREB-LUC and serum starved for 18 hours ± 2 µM HG-9-91-01. CREB-LUC was quantified in cell lysates (**E**). Asynchronously proliferating cells were stimulated with 2 µM HG-9-91-01 for 24 hours with BrdU present for the final 6 hours (**F**). VSMC were transfected with scrambled siRNA (siNEG) or siRNA against CRTC2 and CRTC3 (siCRTC2/3) and grown in 5% FCS for 24 h. Cells were treated with 1 µg/ml BAY60-6583 (BAY) for 24 h with BrdU present for the final 6 h to label cells (G, n = 4). VSMC were transfected with siNEG or siRNA against CREB. Cells were then infected with Ad:control or Ad:CRTC2. Proliferation was quantified 24 hours later by labelling with BrdU for 6 hours (n = 4; **H**).

In a real-time scratch wound migration assays, neither CRTC2 nor CRTC3 affected the rate of VSMC migration over 24 hours (Supplement Fig. 4). However, expression of either active-CRTC2 or active-CRTC3 significantly decreased proliferation in an asynchronous population of VSMC as determined by BrdU incorporation (40.5 ± 4.0% BrdU positive compared to 21.8 ± 4.5% and 30.6 ± 4.2% for CRTC2 and CRTC3 respectively, p < 0.05, Fig. 6A). Moreover, after serum starvation, active CRTC2 significantly decreased serum-induced proliferation (from 40.1 ± 3.8 to 10.0 ± 3.5%) or platelet derived growth factor (PDGF_BB_)-induced proliferation (from 28.5 ± 1.5 to 12.9 ± 1.4%, both p < 0.001, Fig. 6B and C). Active CRTC3 significantly decreased serum-induced proliferation (40.1 ± 3.8 to 24.5 ± 3.7%, p < 0.05) and resulted in a small decrease in PDGF_BB_-induced proliferation that was not statistically significant (Fig. 6B and C). We next employed pharmacological inhibition of SIK, using the highly selective SIK inhibitor HG-9-91-01^37^ to activate endogenous CRTC proteins. SIK-mediated phosphorylation of CRTC proteins is reported to induce their cytoplasmic sequestration by 14-3-3ε and thus their inactivation^38^. Consistent with this, SIK inhibition with HG-9-91-01 reduced cytoplasmic levels of endogenous CRTC2 and CRTC3 and induced their accumulation in the nuclear fraction (Fig. 6D). HG-9-91-01 treatment also significantly increased CREB activity (Fig. 6E) and significantly inhibited VSMC proliferation (Fig. 6F).

To further investigate the role of CRTC2 and CRTC3 in mediating the anti-mitogenic effects of cAMP-elevating signals, we tested the ability of the adenosine A2B receptor agonist BAY60-6583 to inhibit proliferation of VSMC in which both CRTC2 and CRTC3 had been silenced. In siNegative transfected cells, treatment with BAY60-6583 resulted in a significant inhibition of BrdU incorporation (from 29.36 ± 1.70 to 14.03 ± 3.00%; p < 0.01 n = 4; Fig. 6G). In siCRTC2/3 transfected cells, basal proliferation rates were not significantly different from siControl transfected cells (29.36 ± 1.70 vs 29.11 ± 2.76% respectively). However, BAY60-6583 stimulation did not significantly inhibit proliferation in siCRTC2/3 transfected cells and proliferation rates after BAY60-6583 stimulation were significantly lower in siControl transfected cells compared to CRTC2/3 silenced cells (Fig. 6G). Taken together, these data demonstrate that CRTC2 and CRTC3 contribute to the anti-mitogenic effects of cAMP in VSMC. Finally, we tested if these anti-mitogenic effects of CRTC2 and CRTC3 are mediated via activation of CREB, given that CRTC proteins have also been reported to interact with several other transcription factors^39,40^. To this end, we tested the ability of active CRTC2 to inhibit proliferation in VSMC where CREB was silenced. Adenovirus-mediated expression of constitutively active-CRTC2 significantly inhibited proliferation in siControl transfected cells (Fig. 6H). Importantly, constitutively active-CRTC2 did not affect proliferation in CREB-silenced cells, indicating the CREB is essential for the anti-mitogenic effects of CRTC2 (Fig. 6H). Interestingly, CREB silencing also significantly reduced the basal proliferation rate of Ad:Control infected cell.

### Mitogen induced CREB-activity is CRTC-independent, associated with increased serine-133 phosphorylation and required for VSMC proliferation

**Figure 7:**
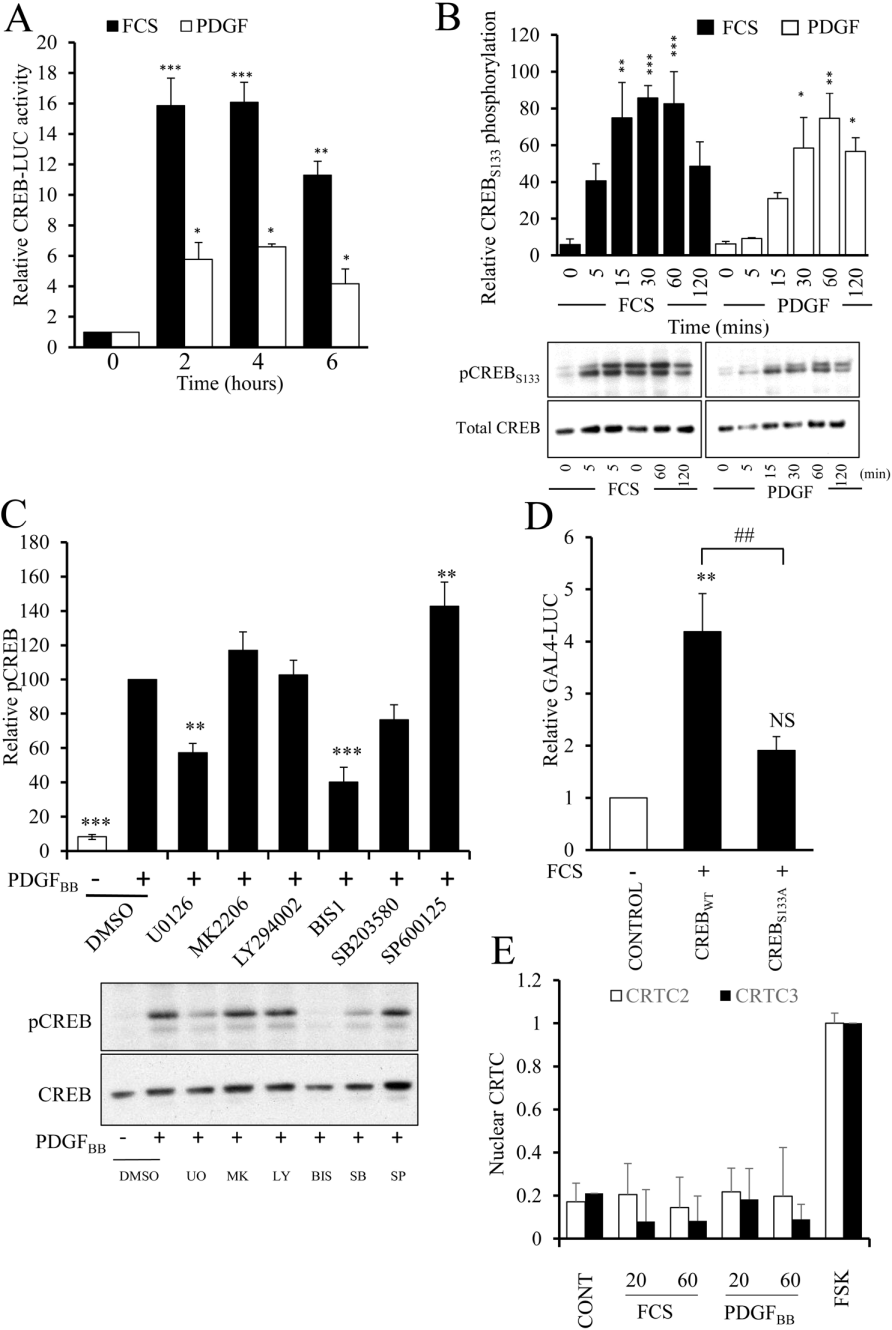
Mitogen induce activation of CREB is serine-133 phosphorylation dependent but CRTC independent. VSMC were transfected with CREB-LUC and serum starved for 24 hours. Cells were stimulated with 5% serum or 25 ng/ml PDGF_BB_ for the indicated times and luciferase activity quantified (n = 3; **A**). VSMC serum starved for 4 h were stimulated for 5, 15, 30, 60 or 120 min with 5% FCS or 25 ng/ml PDGF_BB_ (**B**) or pre-treated with UO126 (UO, 10 µM), MK-2206 (MK, 10 µM), LY294002 (LY, 10 µM), Bisindolylmaleimide I (BIS1, 5 µM), SB 203580 (SB, 10 µM) or SP600125 (SP, 10 µM) and then stimulated with PDGF for 30 min (**C**). Phosphorylated CREB was detected in cell lysates by western blotting with a pCREB (S133) antibody, membranes were reprobed with a total CREB antibody. Phospho-CREB bands were quantified by densitometry and expressed relative to the maximum value in each individual experiment. VSMC were transfected with GAL4-NLUC together with either a GAL4, GAL4-CREB_wild-type_ or GAL4-CREB_S133A_ expression vector and serum starved for 24 hours before stimulation with 5% serum for 4 hours (n = 5; **D**). VSMC (n = 6) serum starved for 4 h were treated for 20 or 60 min with 5% FCS or 25 ng/ml PDGF. Cells were subjected to fractionation and nuclear extracts analysed by Western blotting and densitometry for CRTC2 and CRTC3 (**E**). *Indicates p < 0.05, **indicates p < 0.01, ***indicates p < 0.001 relative to non-stimulated; one-way repeated measures ANOVA with Student Newman Keuls post-hoc tests.

We next investigated if mitogenic stimuli also induced CREB-dependent transcription and if this was mediated by CRTC activation or increased CREB serine-133 phosphorylation. Stimulation with 5% serum significantly increased CREB activity after 2 hours and this persisted for at least 6 hours (Fig. 7A). Stimulation with PDGF_BB_ also significantly increased CREB activity after 2, 4 and 6 hours, although to a lesser extent than that induced by 5% serum. Stimulation with 5% serum resulted in a rapid and significant increase in CREB phosphorylation at serine-133 within 15 minutes (p < 0.01 at 15 min) and this persisted for 60 minutes (Fig. 7B). PDGF_BB_ stimulation also significantly induced CREB phosphorylation at serine-133 but this was slightly delayed compared to serum stimulation, being significant after 30 minutes and persisting for 2 hours (Fig. 7B). To determine which kinases are involved in mediating mitogen-induced serine-133 CREB phosphorylation we treated cells with a panel of pharmacological kinase inhibitors of mitogen-regulated kinases. Inhibition of AKT (using MK2206), PI3-Kinase (using LY294002) or p38 MAPK (using SB 203580) did not significantly affect CREB phosphorylation at serine-133 (Fig. 7C). Inhibition of JNK (using SP600125) resulted in a small but significant increase in CREB phosphorylation. However, inhibition of MEK (using U0126) or conventional PKCs (using bisindolylmaleimide I) strongly and significantly inhibited CREB phosphorylation in response to PDGF_BB_ stimulation (Fig. 7C). To determine if mitogen induced activation of CREB is dependent on serine-133 phosphorylation we tested the ability of serum to stimulate the activity of a GAL4 reporter gene in cells expressing either GAL4-CREB_wild-type_ or GAL4-CREB_S133A_, in which serine-133 is mutated to an alanine. Serum stimulation resulted in a significant induction of GAL4 reporter activity in cells expressing GAL4-CREB_wild-type_ but not in cells expressing GAL4-CREB_S133A_ (Fig. 7D). We also tested if mitogen induced activation of CREB is associated with activation of CRTC2 or CRTC3. Serum or PDGF_BB_ stimulation did not result in any detectable increase in nuclear levels of endogenous CRTC2 or CRTC3 (Fig. 7E and Supplement Fig. 5). Together, these data imply that induction of CREB activity by mitogens, in contrast to cAMP elevating stimuli, is dependent on MEK and PKC dependent phosphorylation of CREB at serine-133 but independent of CRTC2 and CRTC3 activation.

### CREB activity contributes to mitogen-stimulated proliferation of VSMC

**Figure 8:**
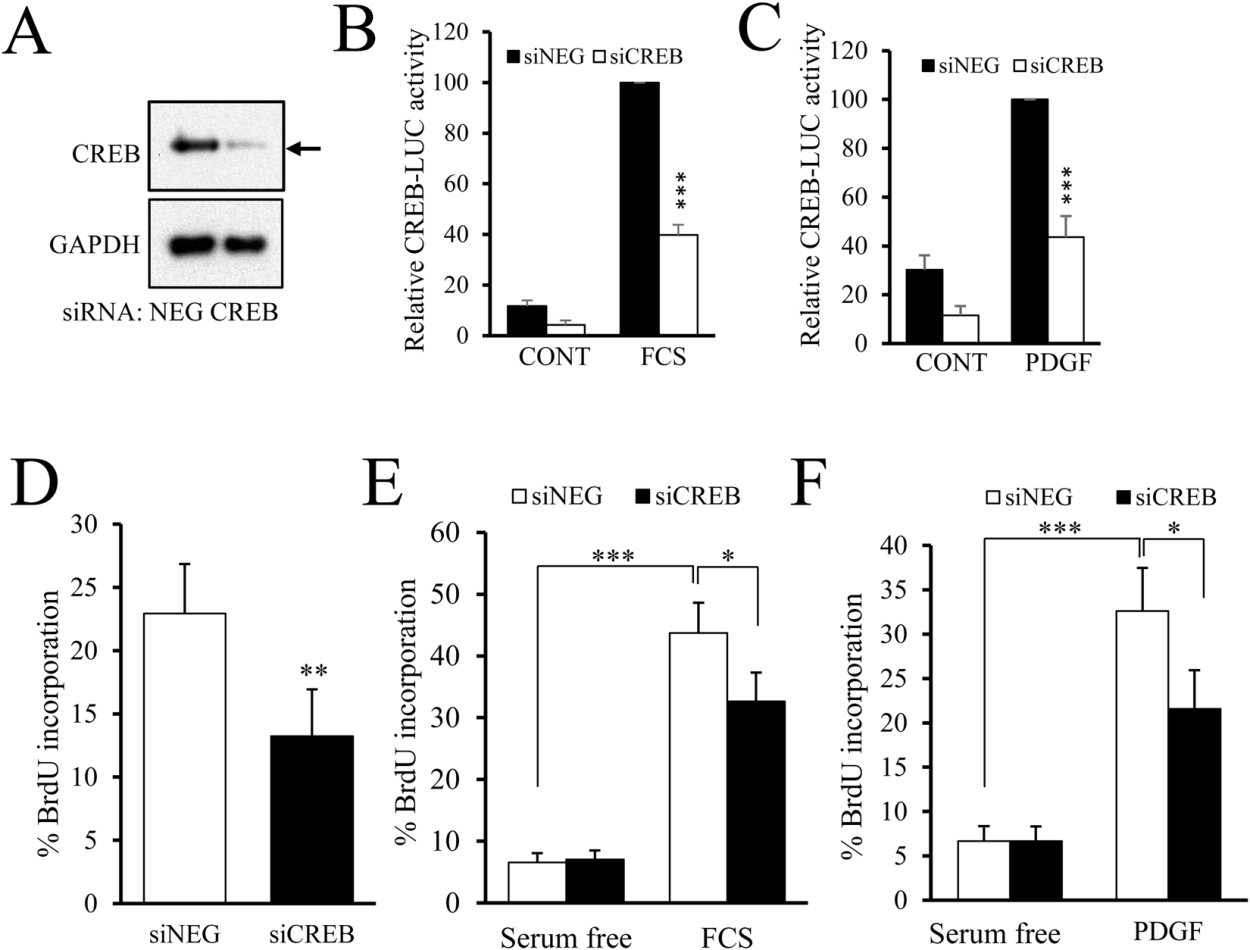
CREB activity contributes to mitogen-stimulated proliferation of VSMC. VMSC were transfected with control siRNA (siNEG) or siRNA targeting CREB (siCREB) and knockdown verified by western blotting after 48 h using anti-CREB and GAPDH antibodies (**A**). Cells were co-transfected with CREB-LUC and siNEG or siCREB. 24 h after transfection cells were serum starved for 3 h, followed by stimulation with 5% FCS (**B**, n = 6) or 25 ng/ml PDGF_BB_ (**C**, n = 6) for 4 h and luciferase activity measured. Asynchronously proliferating VSMC were transfected with siNEG or siCREB. 24 hours post-transfection, cells were incubated with 10 µM BrdU (**D**, n = 5); or serum starved for 30 h then stimulated with 5% FCS (**E**, n=) or 25 ng/ml PDGF_BB_ (**F**, n=) for 24 h, with BrdU present during the final 6 h to label cells. Cells transfected with siNEG or siCREB for 24 h were subsequently infected with Ad:control or Ad: CRTC2 for 24 h and incubated with BrdU for 6 h to label proliferating cells (**G**). *Indicates p < 0.05, **indicates p < 0.01, ***indicates p < 0.001; one-way repeated measures ANOVA with Student Newman Keuls post-hoc tests.

To test whether CREB activity is required for mitogen-stimulated VSMC proliferation, we silenced CREB in VSMC using siRNA. Transfection with CREB targeting siRNA reduced CREB protein levels below the limit of detection by Western blotting without affecting levels of GAPDH protein (Fig. 8A). This reduction in CREB protein expression was associated with a significant decrease in serum- and PDGF_BB_-mediated CREB reporter activity (Fig. 8B and C, both p < 0.001). CREB silencing also significantly reduced proliferation of an asynchronous population of VSMC as determined by BrdU incorporation (Fig. 8D, 22± to 13±% in siNEG versus siCREB, p < 0.01). Moreover, CREB siRNA inhibited both serum- (Fig. 8E) and PDGF-induced (Fig. 8F) increases in proliferation (40± to 33±% and 32± to 23±% respectively, both p < 0.05). These data demonstrate that mitogen-stimulated activation of CREB, which is dependent on serine-133 phosphorylation, is required for efficient VSMC proliferation.

**Figure 9:**
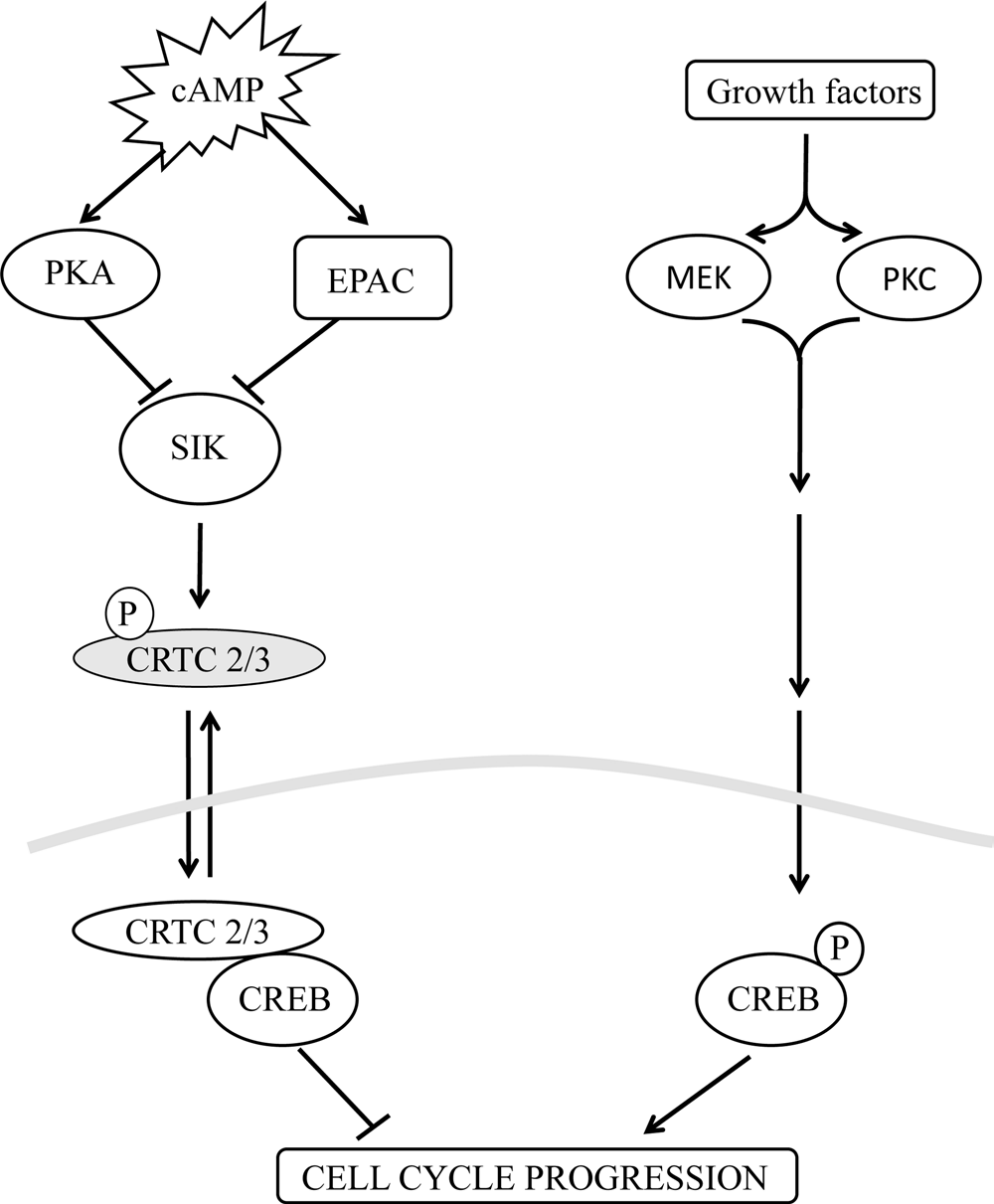
Schematic representation illustrating the mechanisms underlying the pro- and anti-mitogenic functions of CREB in VSMC. Stimuli that elevate cAMP activate PKA and EPAC synergise to induce nuclear translocation of CRTC2 and CRTC3 and activate of CREB. This occurs independently of CREB phosphorylation at serine-133 and contributes towards the anti-mitogenic properties of cAMP in VSMC. Growth factors also induce CREB activation. This is dependent on increased phosphorylation of CREB at serine-133 but independent of CRTC2 or CRTC3 activation. This mechanism is regulated by MEK and PKC and is pro-mitogenic.

## Discussion

In this study, we investigated the role played by CREB phosphorylation at serine-133 and the CREB co-factors, CRTC2 and CRTC3, in mediating the pro- or anti-mitogenic effects of elevated cAMP in vascular smooth muscle cells. We demonstrated that cAMP elevating signals can activate CREB-dependent transcription independently of CREB phosphorylation at serine-133 by inducing nuclear translocation of CRTC2 and CRTC3. Furthermore, this CRTC-mediated activation of CREB is anti-mitogenic. Importantly, we also demonstrate a pro-mitogenic function of CREB in response to serum or PDGF_BB_ stimulation. This is regulated independently of CRTC activation and instead dependent on increased phosphorylation of CREB at serine-133. Our data suggests that CREB plays a dual role in controlling VSMC proliferation and that the mode of CREB activation dictates its pro- or anti-mitogenic function in these cells (Fig. 9).

The physiological effects of increased CREB activity in VSMC have been debated for a long time. Many studies have demonstrated that levels of CREB are down regulated in response to a range of cardiovascular risk factors and mitogenic signals^15,20,41^, suggesting that CREB plays an important role in maintaining normal VSMC physiology and repressing proliferation. For example, levels of CREB are reduced in response to aging, hypertension, hyperlipidaemia^15^ and diabetes^41^. CREB levels negatively correlate with the proliferative capacity of VSMC, being high in differentiated VSMC but reduced in phenotypically modulated cells^20^. Many studies have demonstrated that cAMP-elevating stimuli, which potently activate CREB-dependent-transcription, inhibit VSMC proliferation^1,2,19,22,42^. Consistent with this, forced expression of a constitutively-active CREB fused to the viral activation domain VP16, inhibits VSMC proliferation^20^. Taken together, these observations suggest that elevated levels of CREB protein and activity repress VSMC proliferation. In contrast, there are many reports that CREB can also be activated by mitogenic stimuli in VSMC. For example, CREB is activated in response to angiotensin II or thrombin stimulation and VSMC proliferation in response to these mitogens is CREB-dependent^26,27^. This suggests that CREB plays a dual role in regulating VSMC proliferation and raises the question of how a CREB can control both pro- and anti-mitogenic responses.

The canonical pathway of CREB activation involves phosphorylation of CREB on serine-133 in the kinase-inducible domain^43^. This facilitates interaction with CREB binding protein CBP and has been reported to be required for activation of CREB-dependent gene expression^29^. Elevation of cAMP with forskolin in various cell types induces CREB phosphorylation at serine-133 and increases CREB-dependent transcription^30,44–46^. However, these studies use high concentrations of forskolin (typically 10–25 µM), which induce supra-physiological levels of cAMP in VSMC^4^. Consistent with these findings, we were also able to detect increased serine-133 phosphorylation in VSMC stimulated with 25 µM forskolin (Supplement Fig. 1). However, we demonstrated that low concentrations of forskolin (0.25–0.5 µM) or physiological GPCR agonists induce CREB-dependent transcription in VSMC without increasing CREB phosphorylation at serine-133, suggesting that a non-canonical pathway controls cAMP-induced CREB activity in these cells. We demonstrated that these physiological cAMP signals induce CREB activation by stimulating the nuclear translocation of the CREB co-factors, CRTC2 and CRTC3, whereas CRTC1 did not translocate to the nucleus in response to cAMP elevation. Stimulation with low concentrations of forskolin, Cicaprost, adenosine or the adenosine A2B-receptor agonist BAY60-6583, all stimulated nuclear translocation of CRTC2 and CRTC3, without increasing CREB serine-133 phosphorylation. Furthermore, experiments with selective activators of PKA and EPAC demonstrated that these cAMP-sensitive pathways act synergistically to induce CRTC2/3 activation and CREB activity, mirroring their synergistic action inhibiting VSMC proliferation^2^. To our knowledge this is the first time that EPAC has been linked to the regulation of CRTCs or CREB activation. The precise mechanism underlying this regulation remains unclear. However, it is unlikely to involve EPAC-dependent modulation of PKA activity because we previously demonstrated that EPAC activation does not enhance PKA-mediated phosphorylation of VASP or kemptide in these cells^2^. Expression of constitutively active CRTC2 or CRTC3 activated CREB whereas siRNA-mediated CRTC silencing completely blocked forskolin or GPCR-agonist induced CREB activity, demonstrating that CRTC activation was essential for CREB-dependent transcription in response to cAMP elevating stimuli in VSMC.

To the best of our knowledge, nothing has previously been reported regarding the function of CRTCs in VSMC. In other cell types, activation of CRTCs have been linked to various cellular functions including regulation of gluconeogenesis, lipid metabolism, myogenic gene expression^47^, and fasting-dependent long term memory^48^. Here, using constitutively active mutants of CRTC2 and CRTC3, siRNA-mediated silencing and pharmacological inhibition of SIK, we demonstrate for the first time that activation of CRTC2 and CRTC3 inhibits VSMC proliferation. Interestingly, VSMC migration was not affected by CRTC activation. Furthermore, siRNA-mediated silencing of CRTC2 and CRTC3 reverses the growth inhibitory effects of cAMP-elevating stimuli, linking CRTC activation to the anti-mitogenic effects of cAMP in VSMC. This is in contrast to β-cells, where CRTC2 promotes proliferation, suggesting cell-type specific differences in CRTC function^49^. Although CRTC proteins were initially identified as CREB co-factors^34^, recent reports demonstrate that CRTCs can interact with other transcription factors, including MEIS1A^39^ and STAT3^40^. However, our data showing that CREB-silencing abolishes the anti-mitogenic effects of CRTC2 demonstrates that their anti-mitogenic properties are mediated via their CREB co-factor function in VSMC. Taken together, our data demonstrates for the first time that the anti-mitogenic effects of cAMP-elevating stimuli in VSMC occur independently of CREB serine-133 phosphorylation and are instead mediated, at least in part, via CRTC2 and CRTC3-dependent activation of CREB activity.

Importantly, we also provide evidence that CREB plays a role in promoting VSMC proliferation in response to mitogen stimulation. CREB-activity was increased in response to serum or PDGF_BB_ stimulation. However, this was associated with and dependent on increased phosphorylation at serine-133, in contrast to cAMP induced CREB activation. Moreover, mitogen stimulation did not induce nuclear translocation of CRTC2 or CRTC3. This suggests that mitogens and cAMP employ different mechanisms to activate CREB in VSMC. Importantly, CREB silencing inhibited both basal proliferation and serum or PDGF_BB_ stimulated proliferation. This suggests that CREB plays a dual role in regulating VSMC proliferation and that the mode of CREB activation determines the pro- or anti-mitogenic function. Activation of CREB by CRTC2 or CRTC3 in response to cAMP elevating stimuli occurs independently of CREB serine-133 phosphorylation and promotes growth arrest. In contrast, activation of CREB by in response to mitogenic growth factors is dependent on increased CREB serine-133 phosphorylation, is CRTC independent and is required for efficient proliferation.

The transcription factor CREB is emerging as an important player in the development of vascular disease and intima formation. Our new data highlights the dual role that CREB plays controlling VSMC proliferation and identifies the CRTC-CREB pathway that selectively controls the anti-mitogenic functions of CREB. Future work should focus on determining if this pathway is amenable to pharmacological manipulation, possibly via inhibition of SIK, to exploit the potential vascular protective effects of intervening in this pathway.

## Methods

### Materials

All chemicals were obtained from Sigma unless otherwise stated. Antibodies to CREB (#9104), phospho S133 CREB (#9196), VASP (#3132), CRTC3 (#2720) and Lamin A/C (#4777) were from Cell Signaling Technologies. Antibodies to GAPDH (#MAB374) and CRTC2 (#ST1099) were from Merck-Millipore. Anti-BrdU antibody (B2531) was from Sigma Aldrich. Silencer® Select siRNA targeting CRCT2 and CRTC3 (#4390791 and #4390771 respectively) and negative control (#4390843) were purchased from Thermo Fisher. ON-TARGETplus SMARTpool CREB siRNA (#L-092995-02-0005) and ON-TARGETplus Non-targeting Control Pool (#D-001810-10-05) were purchased from Dharmacon. UO126, LY294002, SB 203580 were from Merck-Calbiochem. Bisindolylmaleimide I (BIS1, GF 109203×) was purchased from Tocris Bioscience. MK-2206 was from Selleckchem. HG-9-91-01 was a gift from Dr Kris Clark (University of Dundee)^37^. Forskolin was purchased from Sigma Aldrich. BAY60-6583^50^ and Cicaprost were purchased from Caymen Chemical. PDGF-BB was purchased from R&D systems. 6-BNZ-cAMP-AM^35^, a cell permeable selective PKA agonist, and 8-pCPT-2′-O-Me-cAMP-AM, a cell permeable selective EPAC super-agonist^36^, were purchased from Biolog.

### Smooth muscle cell culture

All experiments were performed using different batches of cells that were prepared from different animals. Male Sprague Dawley rats were killed by cervical dislocation in accordance with the Directive 2010/63/EU of the European Parliament, under schedule 1 of the United Kingdom Home Office Animal Scientific Procedures Act 1986 and performed in accordance with guidelines, regulations and approval of the University of Bristol. Approval was granted by the University of Bristol ethical review board. Cultures of rat aortic VSMCs (RaVSMCs) were prepared as previously described^51^ Stimulations were performed in serum free DMEM unless otherwise stated. Cells were serum starved for 18 hours or 4 hours, as indicated in figure legends. Proliferation was measured by culture in the presence of 10 µM bromodeoxyuridine (BrdU) for 6 hours. Following fixation in 70% ethanol, incorporated BrdU was detected by immune-histochemical staining as previously described^2^. Typically, all cells (at least 200) in five to ten fields of view were manually counted using ImageJ software. For nuclear and cytosolic fractionation, cells were lysed with ice-cold hypotonic lysis buffer (10 mM HEPES pH 7.8, 10 mM KCl, 1.5 mM MgCl_2_, 1 mM EDTA, 1 × protease (Complete) and phosphatase (PhosTOP) inhibitor mix (Roche Diagnostics Ltd)). After incubation for 20 min on ice with regular vortexing, nuclei were pelleted at 2000 g at 4 °C and the supernatant was collected as the cytosolic fraction. After washing the pellet in lysis buffer containing 0.1% NP40, nuclear proteins were extracted in SDS lysis buffer (1% SDS, 10 mM Tris pH6.8, 10% glycerol).

#### Real-time scratch wound migration assays

Real-time analysis of migration was performed using a IncuCyte® ZOOM live cell imaging system (Essen BioScience) according to the manufacturer’s instructions. Briefly, RaVSMCs were seeded at 2 × 10^4^ cells/well for into ImageLock-96 well plates (Essen Bioscience). Wells were scratched using a WoundMaker® tool and phase contrast images of cell migration into the wounded area acquired hourly for 24 hours. Relative wound confluence was calculated using the Cell Migration Image analysis module of the IncuCyte® ZOOM software.

### Plasmids and Adenoviral Vectors

CREB reporter plasmid (CREB-LUC; α-168) was a gift from Prof Stan McKnight^52^. FLAG-CRTC1, FLAG-CRTC2 and FLAG-CRTC3 plasmids were a gift from Dr Nina Balthasar (University of Bristol). GFP-CRTC-1, -2 and -3 plasmids were constructed by sub-cloning CRTC-1, -2 or -3 cDNAs into pEGFP-N1 expression vector (Clontech). For adenoviruses expressing constitutively active CRTC2 (S171A) or CRTC3 (S162A), cDNAs were cloned into pDC515 (Microbix) and adenoviruses generated as previously described^2^. GAL4-Nano-luciferase plasmid (GLA4-NLUC) was created by sub-cloning the 5xGAL4 binding elements from plasmid pG5E1b-LUC (a gift from Ugo Moens) into the Nhe1 and Xho1 sites of pNL3.3[*secNluc*/minP] (Promega). pcDNAI-GAL4-CREBwild-type and pcDNAI-GAL4-CREB_S133A_ expression vectors were obtained from Addgene (#46769 and #46771 respectively)^30^.

### Quantitative RT-PCR and Western Blotting

Quantification of mRNA and protein levels was performed by RT-qPCR and western blotting respectively, as described previously^2^. Total RNA, extracted using Ambion Pure-Link kits (Thermo Fisher) and was reverse transcribed using QuantiTect RT kit (Qiagen) and random primers. Quantitative PCR was performed using Roche SYBR Green using a Qiagen Roto-Gene Q PCR machine (20′@95 °C; 20′@62 °C; 20′@72 °C). Primers sequences are described in Supplement Table 1. Data were normalised to non-stimulated controls. Western blots were performed using a Mini-Protean II system. Proteins were transferred to PVDF membrane using wet transfer and detected using ECL (Luminata Forte, Millipore) and a digital ChemiDoc imaging system (Bio-Rad).

### Transient transfection and reporter gene assays

CREB activity was determined by quantifying the luciferase reporter activity in cells transfected with CREB-LUC (α-168), which contains 168 bp of the endogenous alpha polypeptide glycoprotein hormones gene (CGA) promoter containing two identical repeats of the 8-bp core CRE^52,53^. Plasmid transfection was performed by electroporation using an Amaxa Nucleofector-1.5. 1 × 10^6^ VSMCs were transfected with 5 μg of DNA or 100 pmoles of siRNA using the standard Nucleofector program A033. Cells were stimulated with the indicated agents 24 hours post transfection followed by lysis in Promega cell culture lysis buffer or for secreted nano-luciferase reporters cell culture media collected and assays using NanoGlow assay system (Promega). Luciferase and Renilla activity were quantified using the dual reporter assay kit (Promega) according to the manufactures instructions using Glomax Discover luminometer (Promega).

### Statistical Analysis

Statistical analysis was performed using two-way ANOVA, one-way ANOVA with Student-Newman-Keuls post-test or where appropriate a paired student’s t-test, as indicated. *Indicates p < 0.05, **indicates p < 0.01, ***indicates p < 0.001.

## Electronic supplementary material

Supplementary Information
