# Supplementary material for: Dual Role of CREB in The Regulation of VSMC Proliferation: Mode of Activation Determines Pro- or Anti-Mitogenic Function
Source: Sci Rep. 2018 Mar 20;8:4904. doi: 10.1038/s41598-018-23199-4 (PMC5861041; doi:10.1038/s41598-018-23199-4)
Supplement: Supplementary file 1 — Supplementary Information [file 41598_2018_23199_MOESM1_ESM.docx]

**DUAL ROLE OF CREB IN THE REGULATION OF VSMC PROLIFERATION:**

***MODE OF ACTIVATION DETERMINES PRO- OR ANTI-MITOGENIC FUNCTION***

**SUPPLEMENTARY MATERIAL**

Claire Hudson^1^, Tomomi E Kimura^1^, ^2^, Aparna Duggirala^1^, Graciela B Sala-Newby^1^, Andrew C Newby^1^ and Mark Bond^1^

^1^Translation Health Sciences, University of Bristol, Research Floor Level 7, Bristol Royal Infirmary, Bristol BS2 8HW

^2^ Present address: [School of Life Sciences](https://www2.warwick.ac.uk/fac/sci/lifesci/), University of Warwick, Coventry, CV4 7AL

Corresponding Author: Dr Mark Bond, Level 7 Queens Building, Bristol Royal Infirmary, University of Bristol, Bristol, U.K. BS2 8HW. Tel: +44 (0)117 3423586

Email: mark.bond@bris.ac.uk.

**Supplement Table 1**

**
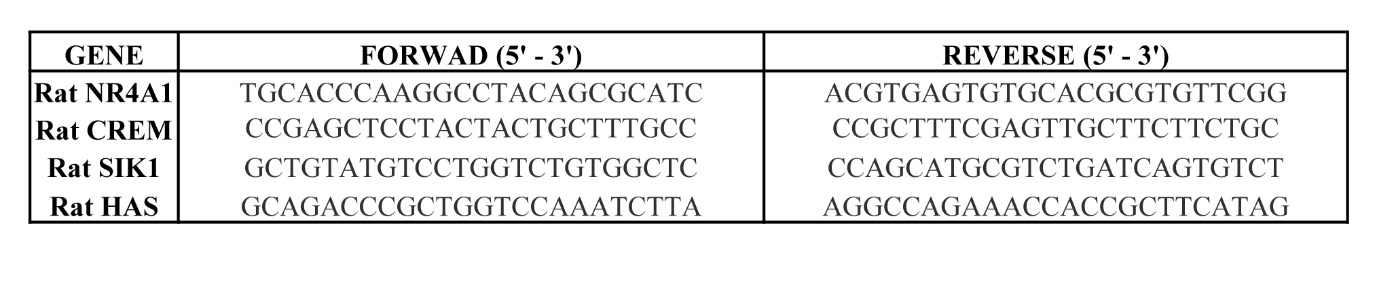
**

**Table 1: Primer sequences used fir qPCR analysis**

**Supplement Figure 1**


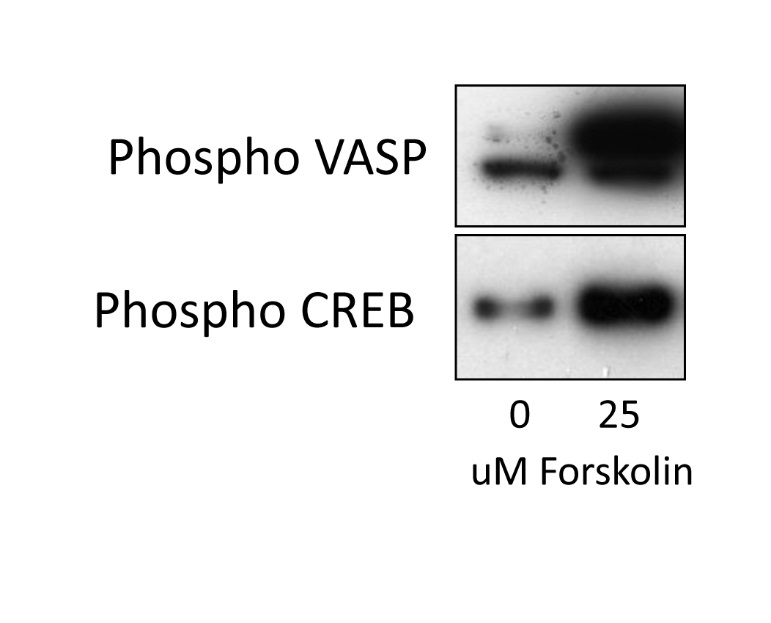


**Supplement Figure 1: Increased CREB phosphorylation in response to 25 µM forskolin stimulation**

Serum starved VSMC were stimulated for 1 hour with 25 µM forskolin for 1 hour. Total cell lysates were analysed by Western blotting for phospho-CREB (S133) and phospho-VASP.

**Supplement Figure 2**

**
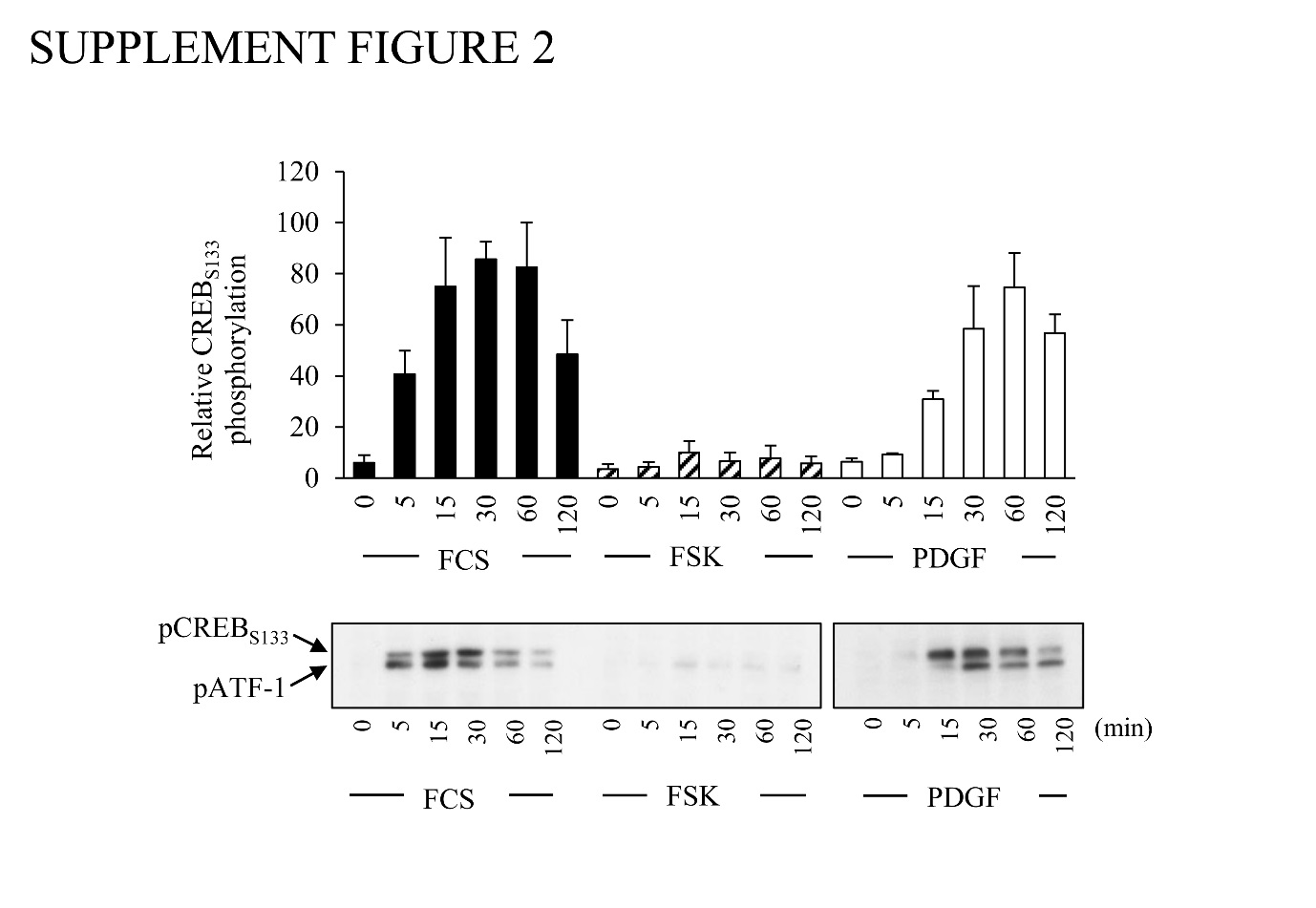
**

**Supplement Figure 2: Time course of CREB phosphorylation in response to serum, 0.5 µM forskolin or PDGF**

Serum starved VSMC were stimulated for 5, 15, 30, 60 or 120 minutes with 5% FCS, 0.5 µM FSK or 25 ng/ml PDGF. Total cell lysates were analysed by Western blotting for phospho-CREB (S133). This antibody also picks up phospho-ATF-1, as indicated.

**Supplement Figure 3
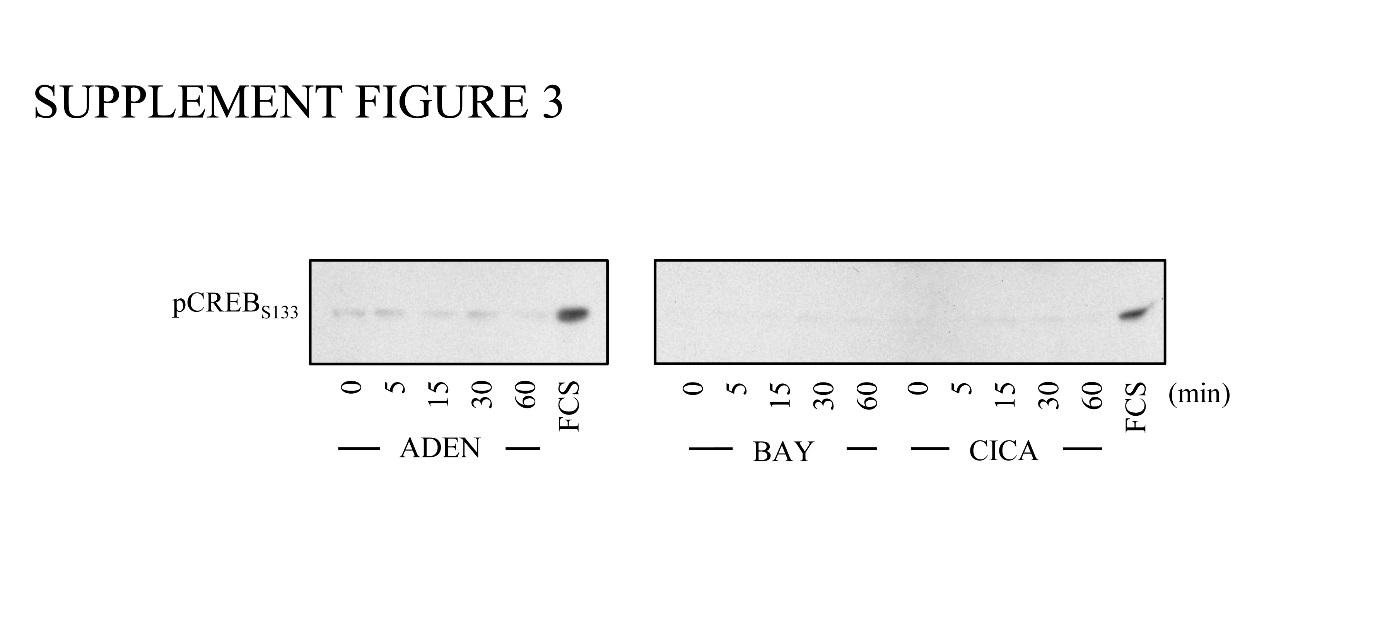
**

**Supplement Figure 3: Time course of CREB phosphorylation in response to adenosine, BAY60-6583 or Cicaprost**

Serum starved VSMC were stimulated for 5, 15, 30 or 60 minutes with 100 µM Adenosine (ADEN), 1 µg/ml BAY60-6583 (BAY), 2 µM Cicaprost (CICA) or 5% FCS. Total cell lysates were analysed by Western blotting for phospho-CREB (S133).

**Supplement Figure 4**


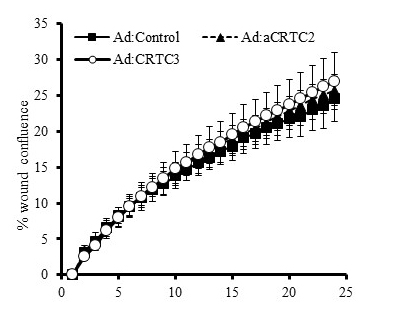


**Supplement Figure 4: Effect of active CRTC2 on VSMC migration**

VSMC (n=3 separate isolates) were infected with either Ad:Control, Ad:aCRTC2 or Ad:aCRTC3 in the presence of 5% serum. 24 hours post infection, migration was quantified using real ®time scratch wound assays using and IncuCyte® ZOOM live cell analysis system.

**Supplement Figure 5**


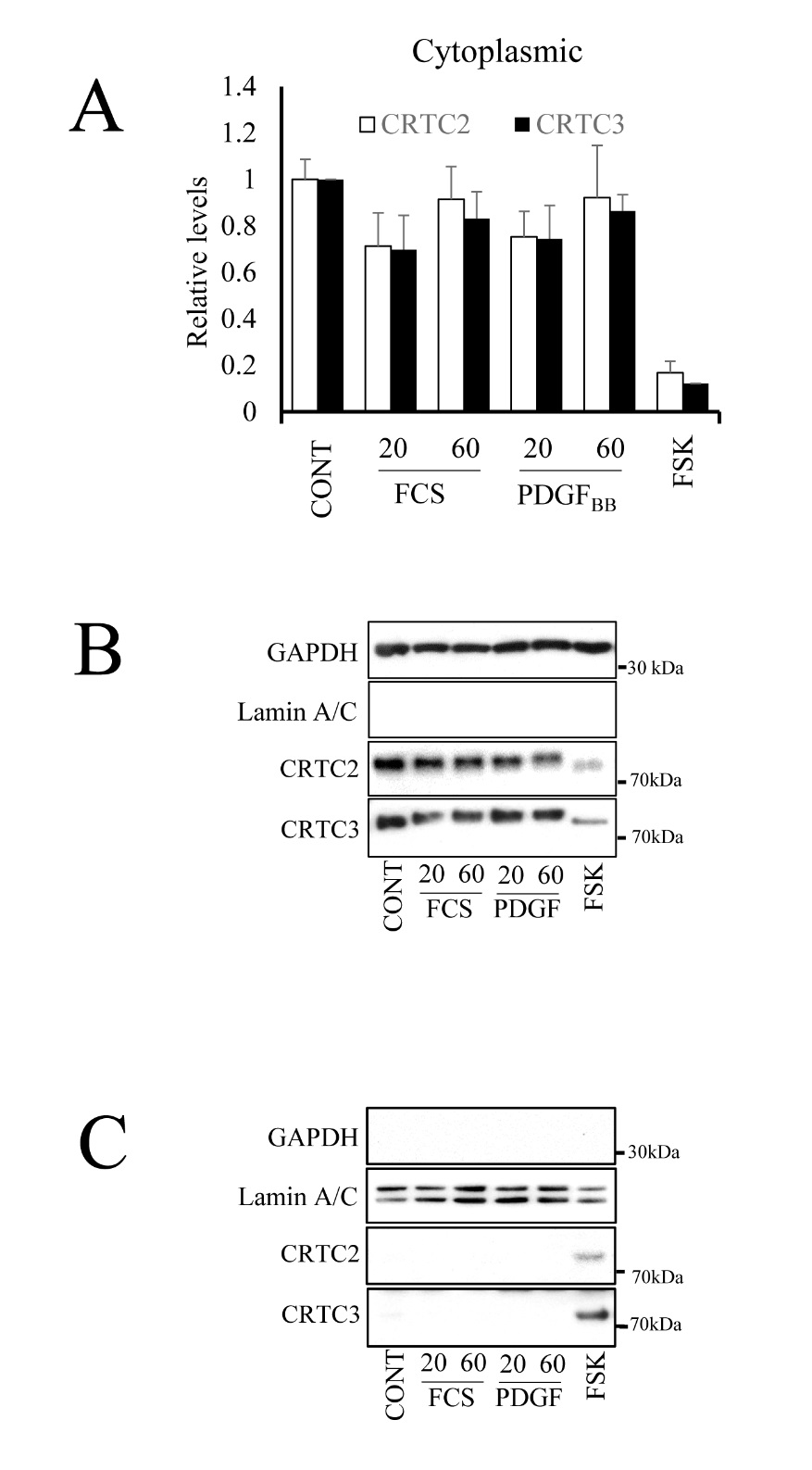


**Supplement figure 5: Effect of serum and PDGF stimulation on CRTC2 and CRTC3 cytoplasmic:nuclear translocation**

VSMC (n=6 separate isolates) serum starved for 4 h were treated for 20 or 60 min with 5% FCS or 25 ng/ml PDGF. Cells were subjected to fractionation and cytoplasmic (A and B) and nuclear (C) extracts analysed by Western blotting and densitometry for CRTC2 and CRTC3.

**ORIGNAL WESTERN BLOT IMAGES USED IN MAIN FIGURES**

**
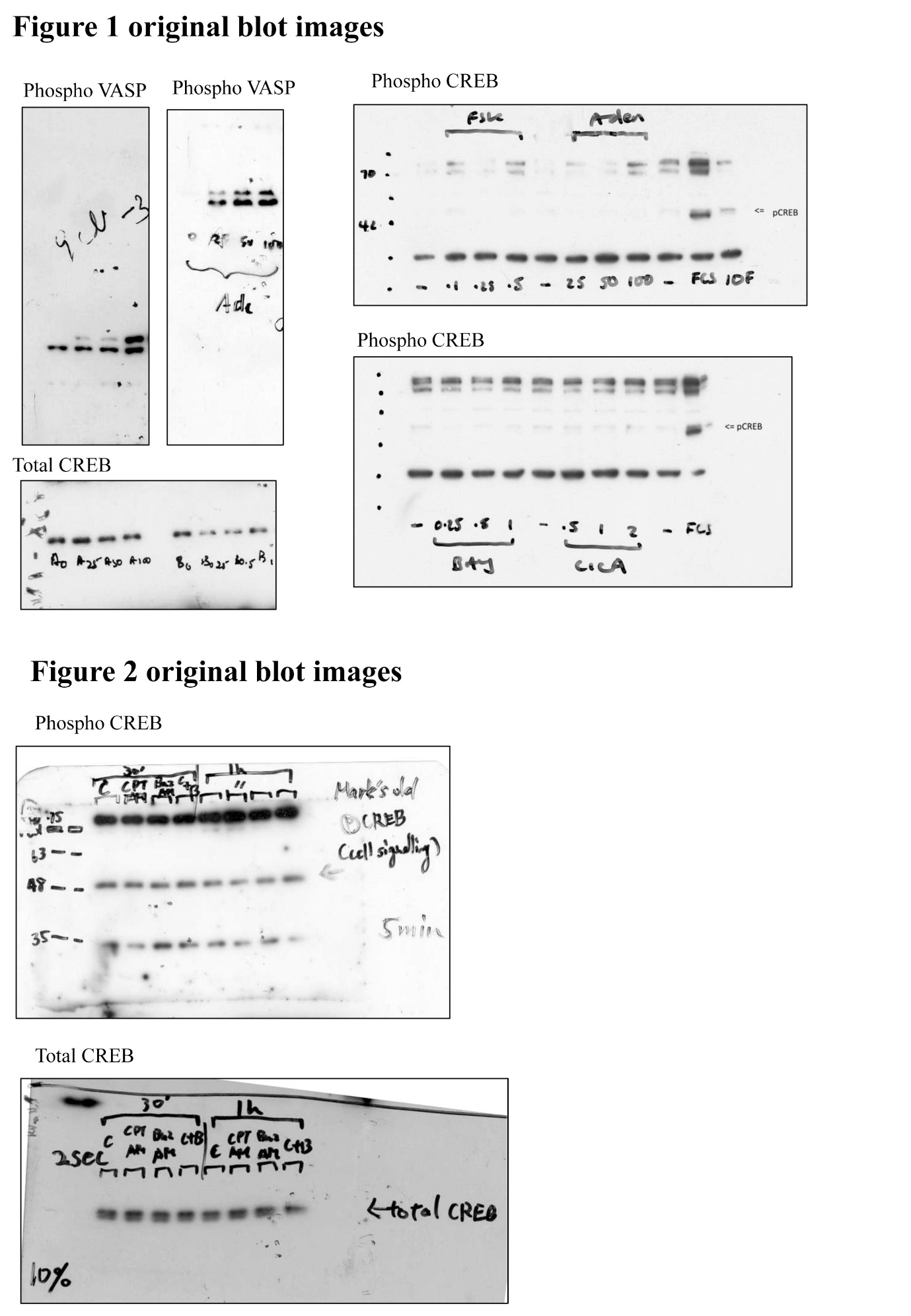

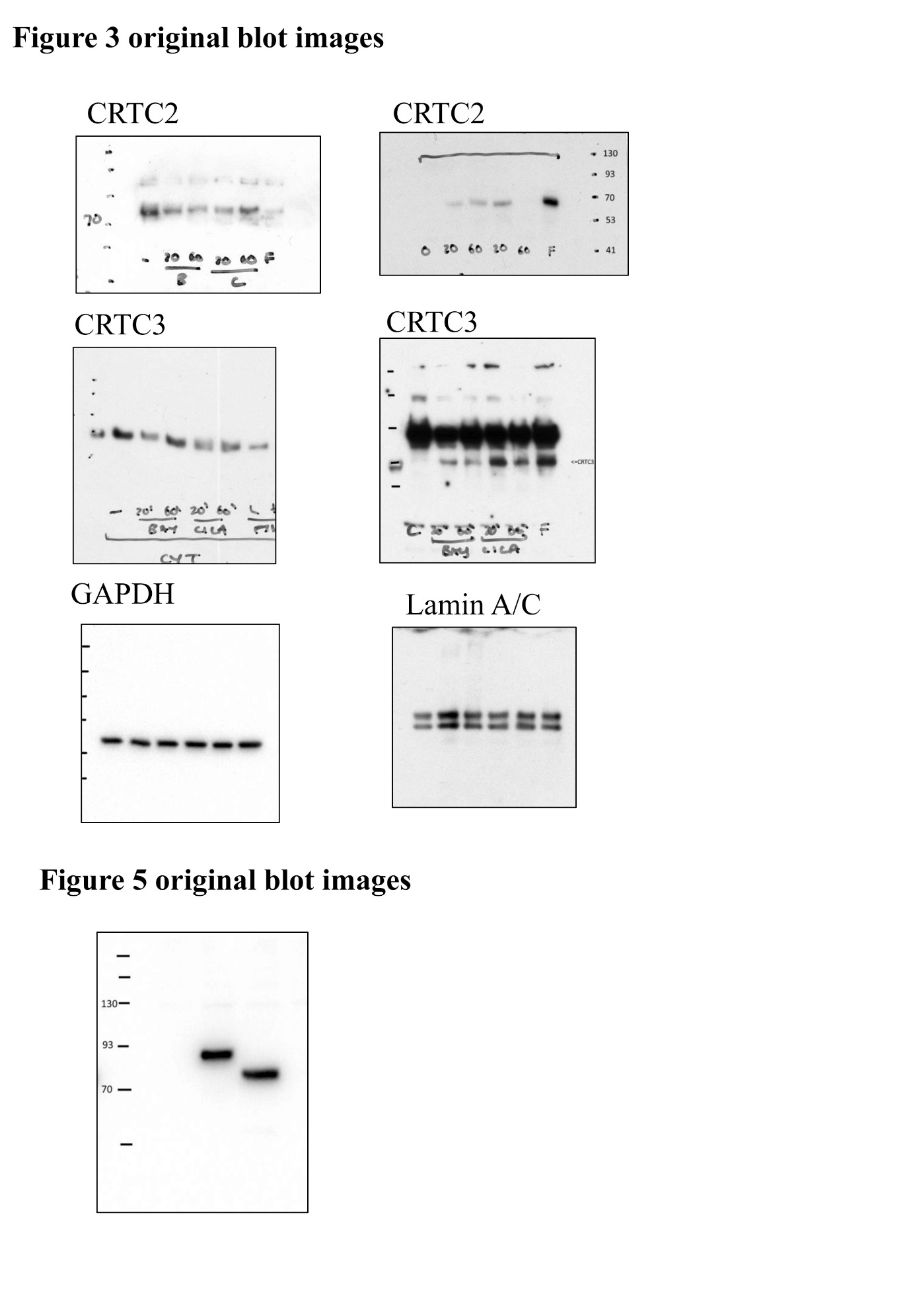
**

**
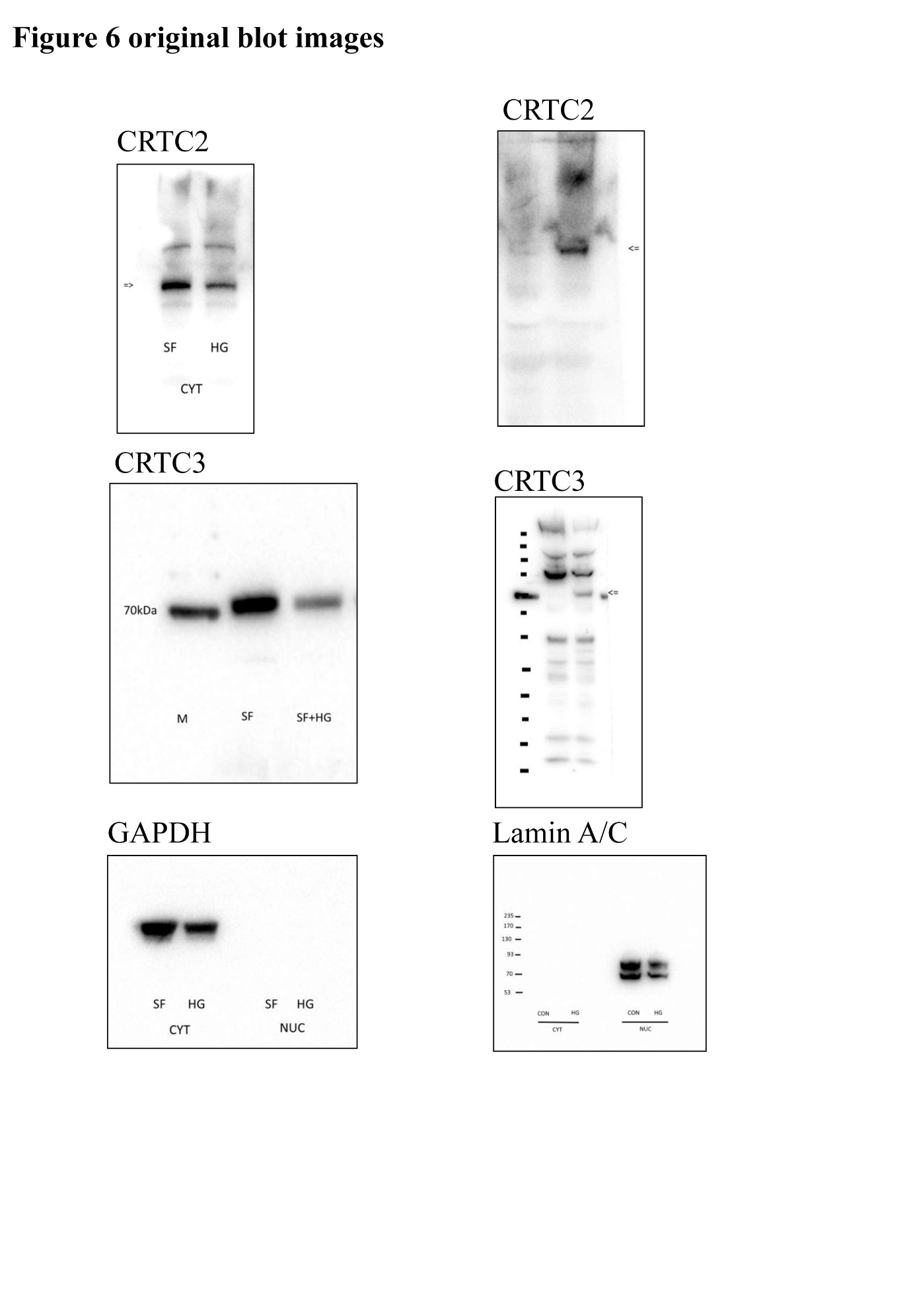

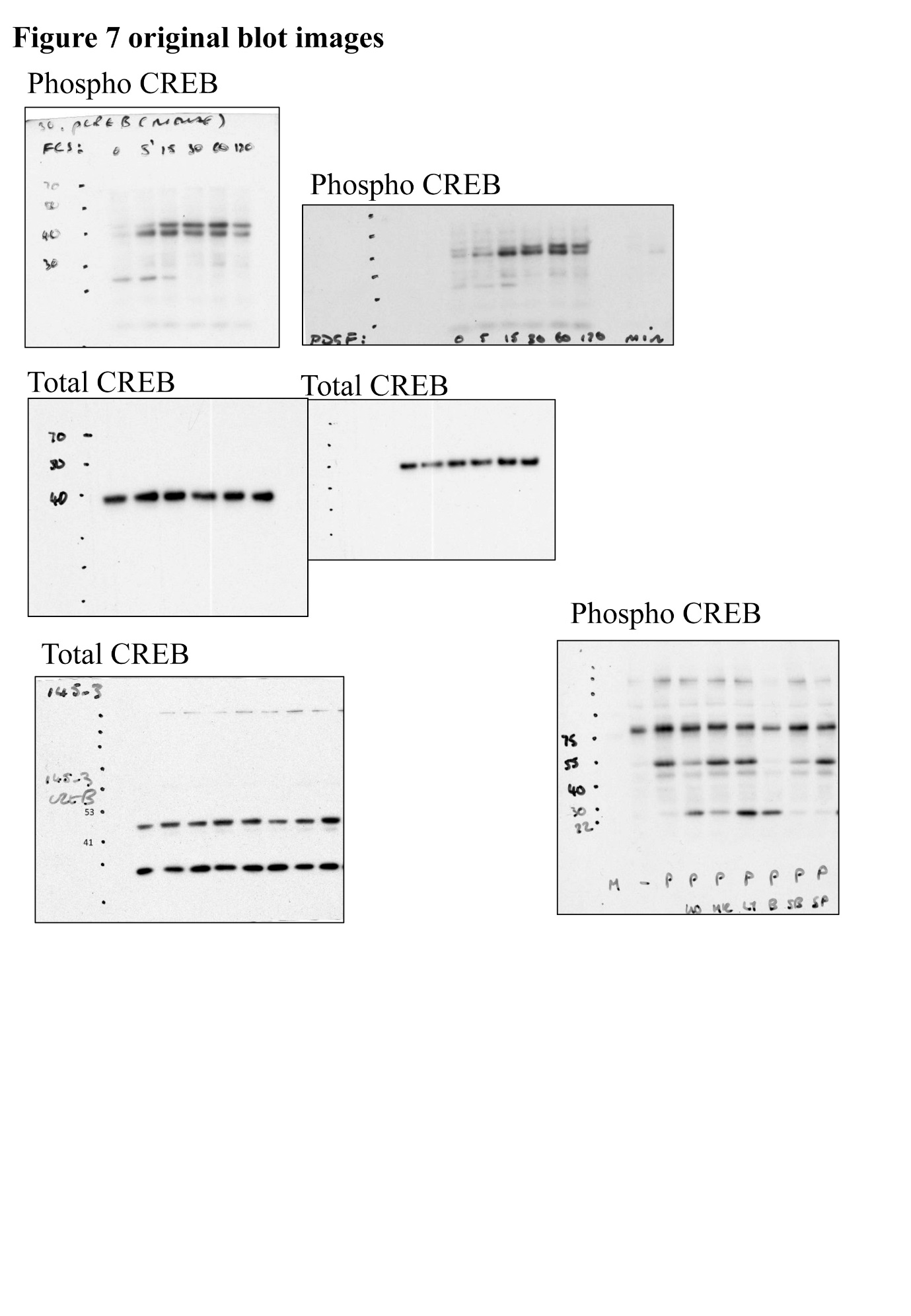
**

**
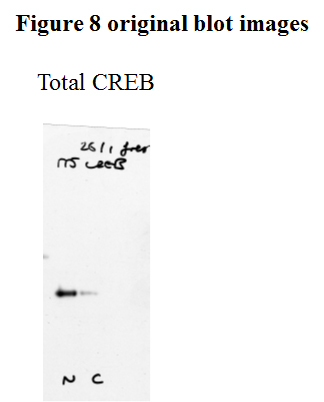
**

**
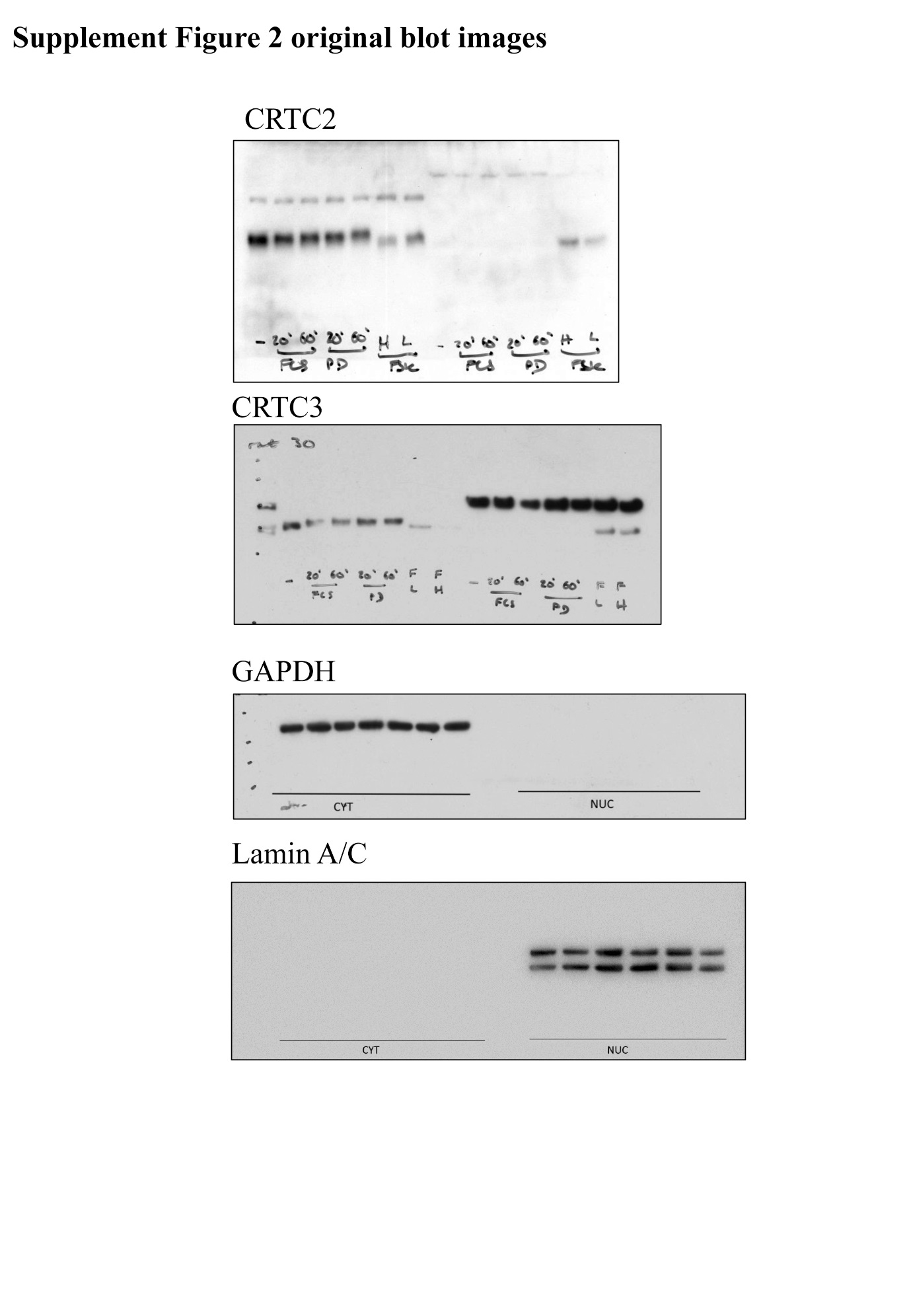
**
